# MSL2 orchestrates oligodendrocyte precursor cell cycle via H3K4me3 to prevent white matter defects in neurodevelopmental disorders

**DOI:** 10.1038/s12276-026-01858-1

**Published:** 2026-10-06

**Authors:** Jie Yuan, Yuhao Dong, Kaixiang Zhang, Zhongcheng Wei, Zhongzhe Zhang, Ming Zhang, Shengjie Luo, Sufang Jiang, Yu Peng, Xinghan Wu, Herenger Yvan, Himanshu Goel, Elaine Zhang, The Australian Undiagnosed Diseases Network (UDN-Aus), Hui Guo, Shengxi Wu, Xianghui Zhao

**Affiliations:** 1 Department of Neurobiology, School of Basic Medicine, Fourth Military Medical University, Xi’an, Shaanxi China; 2 The Shaanxi Province Key Laboratory of Brain Function Analysis and Modulation, Xi’an, Shaanxi China; 3 Center for Medical Genetics, Hunan Key Laboratory of Medical Genetics, MOE Key Lab of Rare Pediatric Diseases, School of Life Sciences, Central South University, Changsha, Hunan China; 4 Department of Medical Genetics, The Affiliated Children’s Hospital of Xiangya School of Medicine (Hunan Children’s Hospital), Central South University, Changsha, Hunan China; 5 Genetica AG, Human Genetics and Genetic Counselling Unit, Zurich, Switzerland; 6 Hunter Genetics, Waratah, NSW Australia; 7 School of Medicine and Public Health, University of Newcastle, Callaghan, NSW Australia; 8 Murdoch Children’s Research Institute, Royal Children’s Hospital, Melbourne, VIC Australia; 9 Department of Paediatrics, University of Melbourne, Melbourne, VIC Australia

**Keywords:** Oligodendrocyte, Experimental models of disease

## Abstract

Pathogenic variants in *MSL2*, a core component of the histone-modifying MSL complex, are associated with neurodevelopmental disorders (NDDs) and white matter abnormalities; however, their mechanisms of pathogenicity remain unknown. In this study, we identified three patients with NDD carrying *MSL2* variants, all of whom exhibited white matter abnormalities. Using conditional knockout mice, we demonstrated that oligodendrocyte lineage-specific deletion of *Msl2* disrupts oligodendrocyte precursor cell proliferation, maturation, and myelination in a cell-autonomous manner, leading to autism-like behaviors including social deficits and repetitive behaviors. Integrated transcriptomic and epigenomic profiling revealed that MSL2 maintains H3K4me3 occupancy at promoters of genes essential for cell cycle progression, mitotic spindle assembly, and myelination, whereas its loss aberrantly activates immune-related pathways. Furthermore, a cell synchronization study confirmed that MSL2 is required for metaphase spindle formation in oligodendrocyte precursor cells, providing a mechanistic basis for impaired oligodendroglial development and hypomyelination. Our findings establish MSL2 as a critical epigenetic regulator of oligodendrocyte lineage progression and propose MSL2-targeted interventions as a promising therapeutic strategy for NDD-related white matter defects.

**Figure:**
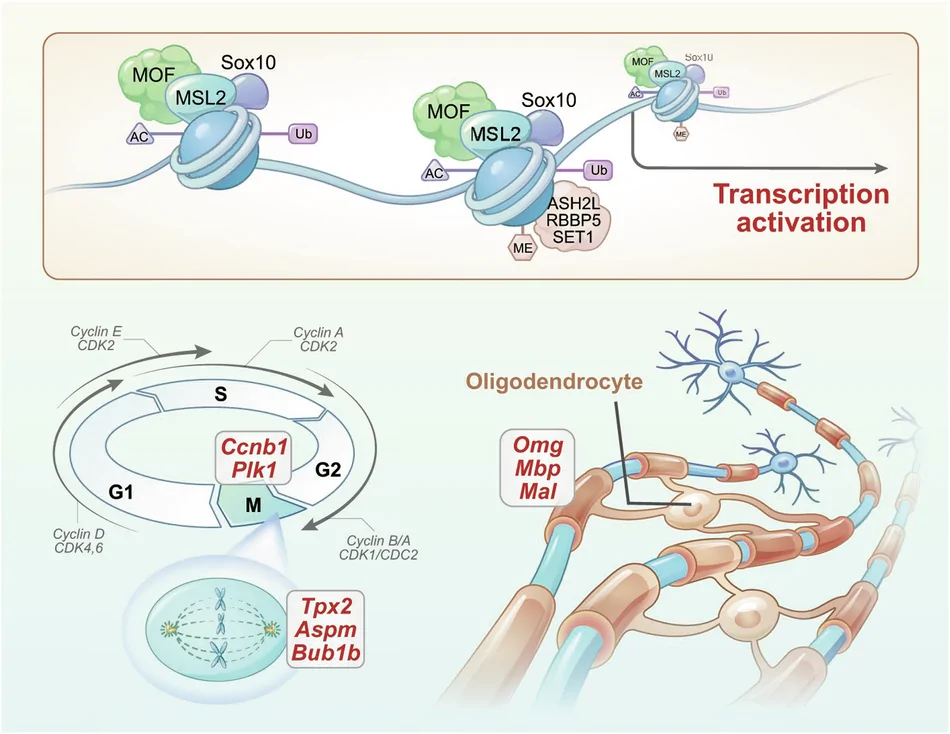

## MSL2 variants disrupt oligodendrocyte development and myelination

Neurodevelopmental disorders (NDDs), including autism and schizophrenia, often involve brain myelination issues. This study explores the role of MSL2, a histone modifier linked to myelination defects, using a mouse model with oligodendrocyte-specific *Msl2* deletion. Researchers found that *Msl2* loss in mice leads to social deficits, anxiety, and depression, mirroring NDD symptoms. The study used genomic and histological methods to show that *Msl2* deletion alters oligodendrocyte precursor cell transcriptomes and histone modifications, affecting cell cycle and differentiation. These findings highlight the critical role of MSL2 in brain development and suggest that targeting MSL2 could offer new therapeutic strategies for NDDs. Future research could focus on its role in neural circuits and its potential in treating demyelination-related disorders.

This summary was initially drafted using artificial intelligence, then revised and fact-checked by the author.

## Introduction

Neurodevelopmental disorders (NDDs) are a group of conditions associated with abnormalities of the neurological system and developmental deficits^1^. Autism spectrum disorder (ASD) and schizophrenia are two major NDDs characterized by overlapping symptoms such as communication difficulties and social withdrawal. These symptoms, originating from altered brain development, can be attributed to maldeveloped myelin in the brain, among other factors^2–4^. Recent genomic studies have identified recurrent de novo variants in *MSL2* (male-specific lethal 2), a histone modifier within the MSL complex, in patients with NDDs exhibiting white matter hypomyelination, including delayed myelination and corpus callosum thinning^5–7^. This clinical link between MSL2 dysfunction and myelination defects highlights the need to explore the role of MSL2 in the cellular process underlying NDD-related white matter abnormalities (WMAs).

Notably, previous investigations into MSL2 function have largely relied on in vitro assays, non-mammalian model organisms (e.g., *Drosophila*), and human cell lines^8–10^. These studies established that MSL2 partners with MOF (KAT8) to catalyze H4 acetylation (H4K16ac), a mark of open chromatin, and acts as an E3 ubiquitin ligase targeting H2B (e.g., H2BK34ub, H2BK120ub), thereby facilitating H3 K4 and K79 methylation via 'trans-tail' crosstalk. All these histone modifications are well-recognized drivers of transcriptional activation. Importantly, the constitutive knockout of *Msl2* in mice is embryonically lethal^11^, which prevents investigation of cell type-specific functions of MSL2, particularly in oligodendrocytes (OLs), the myelinating cells of the brain. The precise role and mechanism of MSL2 in brain development, especially in OL-mediated myelination, remain unresolved.

Myelination by OLs in the brain enables saltatory conduction of action potentials and provides long-term trophic support for axons^12^. The formation of mature myelinating OLs is a complex process tightly coordinated spatially and temporally by genetic and epigenetic events^13,14^. Furthermore, DNA methylation, histone modification, and chromatin remodeling are critical for OL development, function, and regeneration^15^. Given the established role of MSL2 in epigenetic regulation and its association with myelination defects in patients, unraveling its OL-specific function is critical to understand NDD pathogenesis.

To address this key knowledge gap, the core novelty of the present study lies in the generation and analysis of a mouse model with OL-specific depletion of *Msl2*. In this research, we reported on patients with NDDs and novel *MSL2* variants, all of whom show WMAs on magnetic resonance imaging (MRI). Using our OL-specific *Msl2* knockout model, we identified the essential role of MSL2 in OL development. Mice with *Msl2* deletion in the OL lineage develop social interaction deficiency, anxiety, and depression, behavioral phenotypes relevant to NDDs. Moreover, *Msl2* loss alters the transcriptomic profiles of oligodendrocyte precursor cells (OPCs) and results in genome-wide alterations in H3K4me3 modifications, with target genes associated with cell cycle regulation, mitotic spindle formation, and OL differentiation. These data partially explain the observed MRI abnormalities and behavioral consequences in patients with NDDs and *MSL2* variants. We anticipate that our study will open new possibilities for designing cell type-specific intervention strategies against MSL2 in NDDs.

## Materials and methods

### Clinical assessment and molecular genetic methods for *MSL2* families

Three affected individuals harboring *MSL2* variants and their unaffected parents were recruited independently at three clinical centers and connected through GeneMatcher^16^. For each affected individual, detailed clinical information was obtained either through recontact or a comprehensive review of medical records by neurologists, pediatricians, or clinical geneticists. Informed consent was obtained from their parents in compliance with local institutional review board requirements. Ethical approval for this study was granted by the institutional review board of Central South University and the Victorian Royal Children’s Hospital Human Research Ethics Committee (RCH79712). Genomic DNA was extracted from peripheral blood samples of the affected individuals. Families 1 and 2 underwent trio-based exome sequencing, whereas family 3 underwent trio-based whole genome sequencing. In each family, no pathogenic variants were identified other than the *MSL2* variants.

### Animals

All animal experiments were performed according to the National Institutes of Health Guidelines on the Use of Laboratory Animals and approved by the Animal Care and Use Committee of Fourth Military Medical University. *Msl2*^flox/flox^ mice, generated by Cyagen Biosciences, were crossed with Olig1-Cre^17^ or NG2-Cre^ER^ mice^18^ to generate *Msl2* conditional knockout (cKO; *Msl2*^flox/flox^; Olig1-Cre^+/−^) or the OPC-inducible *Msl2* iKO (*Msl2*^flox/flox^; NG2-Cre^ER^) offsprings. To induce Cre expression in *Msl2* iKO mice, 4-hydroxytamoxifen (4-OHT) was dissolved in dimethyl sulfoxide at a stock concentration of 20 mg/ml and injected hypodermically at 3 mg/40 g (body weight) once a day every other day.

### Cell culture and immunocytochemistry

Mouse OPCs were isolated from the P6 cortices of pups, as described previously^19^. OPCs were cultured in Sato medium with PDGF-AA (Peprotech, 100-13 A) and bFGF (Peprotech, 100-18B). CNTF (Peprotech, 450-13), NAC (Sigma, A8199), and T3 (Sigma, T2877) were added to the Sato medium for OL differentiation. Peripheral blood lymphocytes were isolated from freshly collected blood and transformed with Epstein–Barr virus in the presence of cyclosporin A to establish lymphoblastoid cell lines, using a modified protocol based on Neitzel^20^. Cells were maintained in RPMI-1640 supplemented with 25% fetal bovine serum at 37 °C in 5% CO₂. Immunofluorescence staining was performed as before^19^.

### Histological study

For immunohistochemistry, brain cryosections (14 μm) were fixed with 4% paraformaldehyde for 30 minutes, then processed for antigen retrieval with 10 mM sodium citrate (pH 6.0) at 90 °C for 10 minutes, and finally cooled down at room temperature (RT). The sections were washed three times in 1× PBS for 10 minutes each, blocked in a blocking buffer for 1 hour at RT, and then incubated with primary antibodies overnight at 4 °C. The next day, sections were incubated with the corresponding species secondary antibodies at RT for 3 h. Finally, sections were counterstained with DAPI for 5 minutes, sealed with 50% glycerin, and examined on an Olympus FV3000 confocal microscope.

Antibodies used in this study were as follows: SOX10 (Oasis Biofarm OB-PGP001-01, 1:500), PDGFRα (Abcam ab61219, 1:500), CC1 (Sigma OP80, 1:200), MBP (Abcam ab7349, 1:500), CNPase (Sigma C5922, 1:500), MSL2 (Cell Signaling Technology, #4406,1:500), NeuN (Abcam, ab104224, 1:500), MSL2 (Cell Signaling Technology, #4406), H4K16ac (Abcam, ab109463), H3K4me3 (Cell Signaling Technology, #9751), Nuclei (Millipore, MAB1281). Secondary antibodies: donkey anti-rabbit Alexa Fluor 488 (Invitrogen A21206, 1:1000), donkey anti-rabbit Alexa Fluor 594 (Invitrogen A21207, 1:1000), donkey anti-rabbit Alexa Fluor 647 (Invitrogen A31573, 1:1000), donkey anti-guinea pig Alexa Fluor 647 (Jackson ImmunoResearch #706-605-148, 1:500), donkey anti-mouse Alexa Fluor 594 (Invitrogen A21203, 1:1000), and donkey anti-rat Alexa Fluor 488 (Invitrogen A-21208, 1:1000).

For transmission electron microscopy, sample preparations were performed as per the previous study^19^. Ultrathin sections (1 μm) were stained with 2% uranyl acetate and examined with a JEM-1230 electron microscope (JEOL LTD), which is equipped with a CCD camera and its application software (832 SC1000, Gatan). Myelin thickness was quantified by calculating the g-ratio (the ratio between the inner and the outer diameter of the myelin sheath).

### ChIP-seq, CUT&Tag, and data analysis

Chromatin immunoprecipitation (ChIP) assays were performed as previously described^21^. Chromatins from 10^7^ OPCs were sonicated to get soluble sheared chromatin (average DNA length of 200–500 bp). Next, 20 µl chromatin was saved at −20 °C for input DNA, and 100 µl chromatin was used for immunoprecipitation by H3K4me3 antibodies (Cell Signaling Technology, #9751). The resulting immunoprecipitated DNA was used to construct sequencing libraries following the protocol provided by the I NEXTFLEX ChIP-Seq Library Prep Kit for Illumina Sequencing (NOVA-5143-02, Bioo Scientific) and was sequenced on Illumina Novaseq 6000 with the paired-end 150 (PE 150) method. Sequencing service was provided by Wuhan IGENEBOOK Biotechnology Co. Ltd.

For H4K16ac and H2BK120ub profiling, Cleavage Under Targets and Tagmentation (CUT&Tag) assays were performed according to the previous description^22^. Briefly, 10^4^ cell nuclei were harvested and incubated with concanavalin A-coated magnetic beads for 15 minutes at RT. Then, bead-bound cells were resuspended and incubated with the H4K16ac antibody (Abcam ab109463), HBK120ub antibody (Active Motif, 39623), Mouse IgG (Cell Signaling Technology, #68860), and Rabbit IgG (Cell Signaling Technology, #2729) overnight at 4 °C. Subsequently, goat anti-rabbit secondary antibody (Abcam ab206) was added to this mixture for 30 minutes at RT and the magnet stand was used to remove unbound antibodies. The pA–Tn5 adapter complex was added at this step for 1 hour at RT, and the unbound pA–Tn5 protein was removed. Next, cells were resuspended in tagmentation buffer at 37 °C for 1 hour, and the immunoprecipitated DNA was collected. Library construction and sequencing service (on Illumina NovaSeq 6000 with PE 150 method) was provided by Wuhan IGENEBOOK Biotechnology Co. Ltd.

Trimmomatic (version 0.36) was used to filter low-quality reads^23^. Clean reads were mapped to the mouse genome (GRCm39) by BWA (version 0.7.15). Samtools (version 1.3.1) was used to remove potential PCR duplicates. MACS2 software (version 2.1.1.20160309) was used to call peaks by default parameters (bandwidth 300 bp; model fold 5 and 50; *q-*value cutoff = 0.01). If the midpoint of a peak is located closest to the transcription start site (TSS) of one gene, the peak will be assigned to that gene^24^. DiffBind (version 3.4.3) was used for differential peak analysis with a significance threshold of *P* < 0.05 and fold change > 0. The nearest gene to the summit of each differential peak, relative to its TSS, is defined as the corresponding target gene. Furthermore, BEDTools (version 2.1.7) was used to annotate and analyze the distribution of differential peak summits across functional genomic elements. In addition, clusterProfiler (https://bioconductor.org/packages/release/bioc/html/clusterProfiler.html) in the R package was used to perform Gene Ontology (GO) and Kyoto Encyclopedia of Genes and Genomes (KEGG) enrichment analyses, which were calculated using a hypergeometric distribution with a *P-*value cutoff of 0.05^25^. Gene terms highly related to the phenotypes were selected for generating GO enrichment bubble plots. The transcriptome and histone modification profiling datasets have been deposited in NCBI BioProject with the primary accession code PRJNA1272498.

### Statistical analysis

All statistical analyses were performed in Prism v.9.0 (GraphPad Software, Inc.) and SPSS v.21.0 (IBM Inc.). The normality test was performed with the Shapiro–Wilk test, and the homogeneity of variance test was performed by Levene’s test. All data that met normality and homogeneity of variance were compared using a two-tailed unpaired *t*-test and one-way analysis of variance (ANOVA). Datasets that were normally distributed but lacked homogeneity of variance were compared using a two-tailed, unpaired, separate-variance estimation *t*-test. Data that were not normally distributed were analyzed using the Mann–Whitney *U*-test or the Friedman M test. Numerical values were described using mean ± s.e.m. and presented as bar graphs. Significance is denoted as *P < 0.05, **P < 0.01 or ***P < 0.001.

## Results

### WMAs are associated with *MSL2* pathogenic variants

We identified three patients with pathogenic *MSL2* variants. Two harbored putative truncating variants (PTVs)—one de novo frameshift and one stop-gain with undetermined inheritance—and the third carried a de novo microdeletion involving both *MSL2* and *PCCB*. Detailed clinical information is summarized in Supplementary Table 1. In brief, all three patients presented with moderate to severe intellectual disability, language impairment, and motor delay; two patients also exhibited ASD or autistic features. Microcephaly was observed in two patients.

**Fig. 1:**
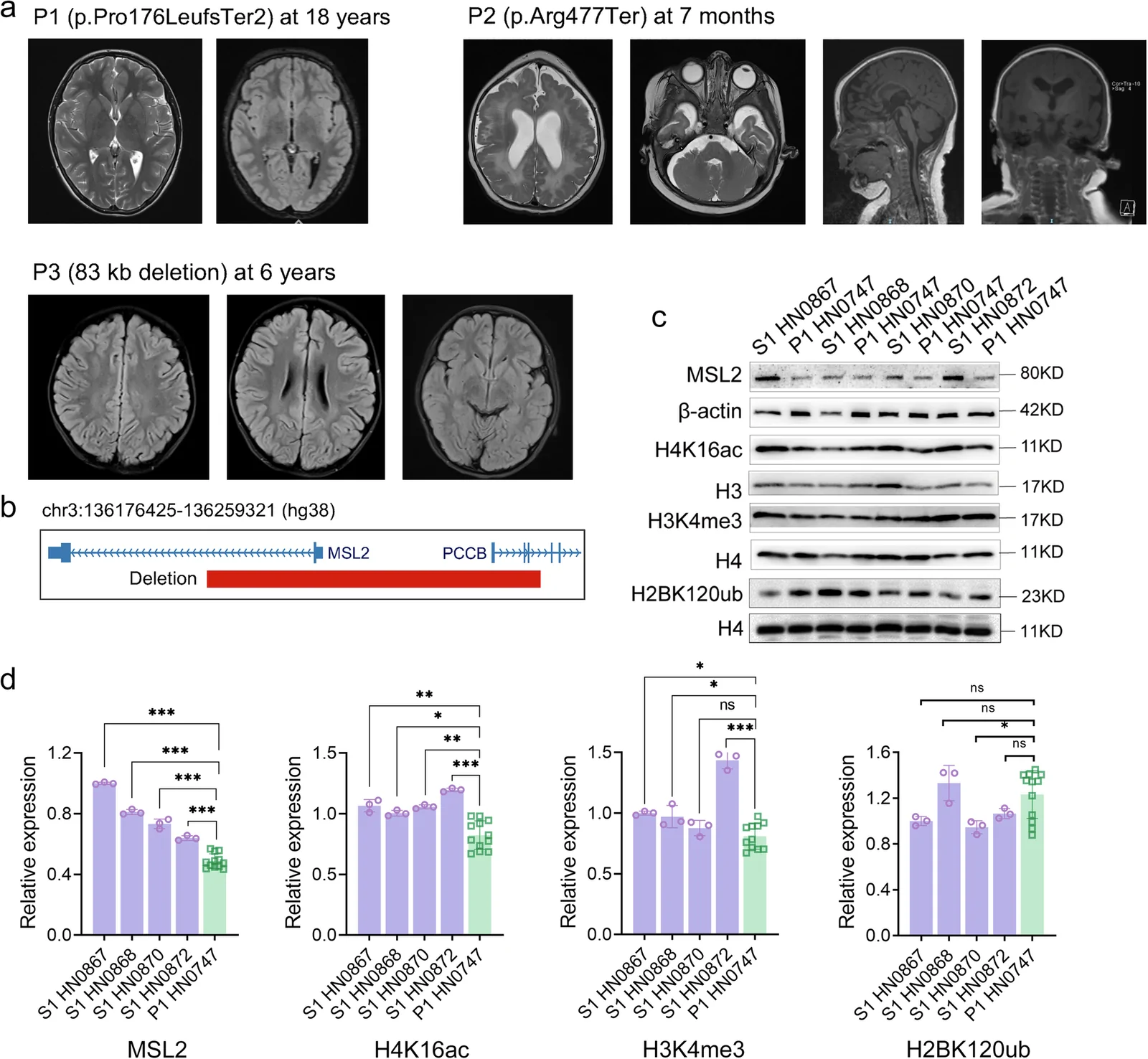
Brain WMAs in individuals with pathogenic *MSL2* variants. **a** MRI images from three patients demonstrate WMAs associated with different *MSL2* mutations. Patient 1 (P1, p.Pro176LeufsTer2), imaged at 18 years of age, shows a small corpus callosum, an intermediate cyst in the pituitary region (pars intermedia), and multiple bilateral, frontally predominant punctate hyperintensities in the white matter. Patient 2 (P2, p.Arg477Ter), imaged at 7 months, exhibits diffuse WMAs, including extensive symmetrical T2 hyperintensity throughout the cerebral hemispheres, reduced cerebral volume with associated ventricular enlargement, abnormal cortical sulcation with widespread bilateral polymicrogyria, and several T1 hyperintense foci indicative of punctate calcifications. Patient 3 (P3), imaged at 6 years, exhibits punctate, slightly hyperintense T2-weighted signals in the bilateral frontal white matter with corresponding hyperintensity on FLAIR, showing ill-defined margins. **b** The panel shows the 83-kb deletion encompassing *MSL2* (chr3:136176425–136259321, hg38) identified in P3. **c**, **d** Western blot analysis and quantification of MSL2, H4K16ac, H3K4me3, H2BK120ub levels in immortalized lymphocytes from P3 (P1 HN0747) and four age- and sex-matched healthy controls (S1 HN0867, S1 HN0868, S1 HN0870, S1 HN0872). β-actin, H3, and H4 were used as loading controls. Data are mean ± s.e.m. (*n* = 3 repeats in control or 12 repeats in P3). Two tail unpaired *t*-test, *P < 0.05, **P < 0.01, ***P < 0.001.

All three patients exhibited WMAs of varying severity (Fig. 1a). Patient 1, an 18-year-old boy with a de novo p.Pro176LeufsTer2 variant, showed a thin corpus callosum, a cyst in the pituitary pars intermedia, and multiple bilateral punctate hyperintensities in the frontal white matter. Patient 2, a 7-month-old boy with a p.Arg477Ter variant with undetermined inheritance, displayed more extensive changes, including diffuse symmetrical T2 hyperintensity throughout the cerebral hemispheres, reduced cerebral volume with ventricular enlargement, abnormal cortical sulcation, widespread bilateral polymicrogyria, and T1 hyperintense foci consistent with punctate calcifications. Patient 3, a 6-year-old carrying a heterozygous 83-kilobase (kb) deletion involving *MSL2*, presented with punctate T2 hyperintensities in the bilateral frontal white matter, corresponding fluid-attenuated inversion recovery(FLAIR) hyperintensity, and ill-defined margins (Fig. 1a,b). Together, these MRI findings underscore the consistent association of WMAs with *MSL2*-related NDDs.

Immortalized lymphocyte cell lines were available from patient 3, allowing experimental evaluation of the functional consequences of the *MSL2* variant. We assessed MSL2 protein level and downstream epigenetic modifications (H4K16ac, H3K4me3, H2B120ub) in immortalized lymphocytes from patient 3 and four age- and sex-matched healthy controls. Both western blotting analysis and immunofluorescence staining demonstrated markedly reduced MSL2 protein levels in patient cells compared with controls (Fig. 1a,b and Supplementary Fig. 1). For H4K16ac and H3K4me3, both assays revealed a downward trend in the patient’s cells relative to most controls, with mild interindividual variability observed among the healthy control group. For H2BK120ub, three out of four healthy controls showed levels comparable to the patient’s cells in the western blotting assay (Fig. 1c,d), suggesting greater physiological variability of this histone modification in the normal population. Collectively, these results verify that the MSL2 variant in patient 3 is associated with impaired MSL2 function and reduced levels of MSL2-regulated H4K16ac and H3K4me3. Patient-derived samples from patients 1 and 2 were unavailable for similar analyses.

### *Msl2* deficiency impairs OL differentiation

**Fig. 2:**
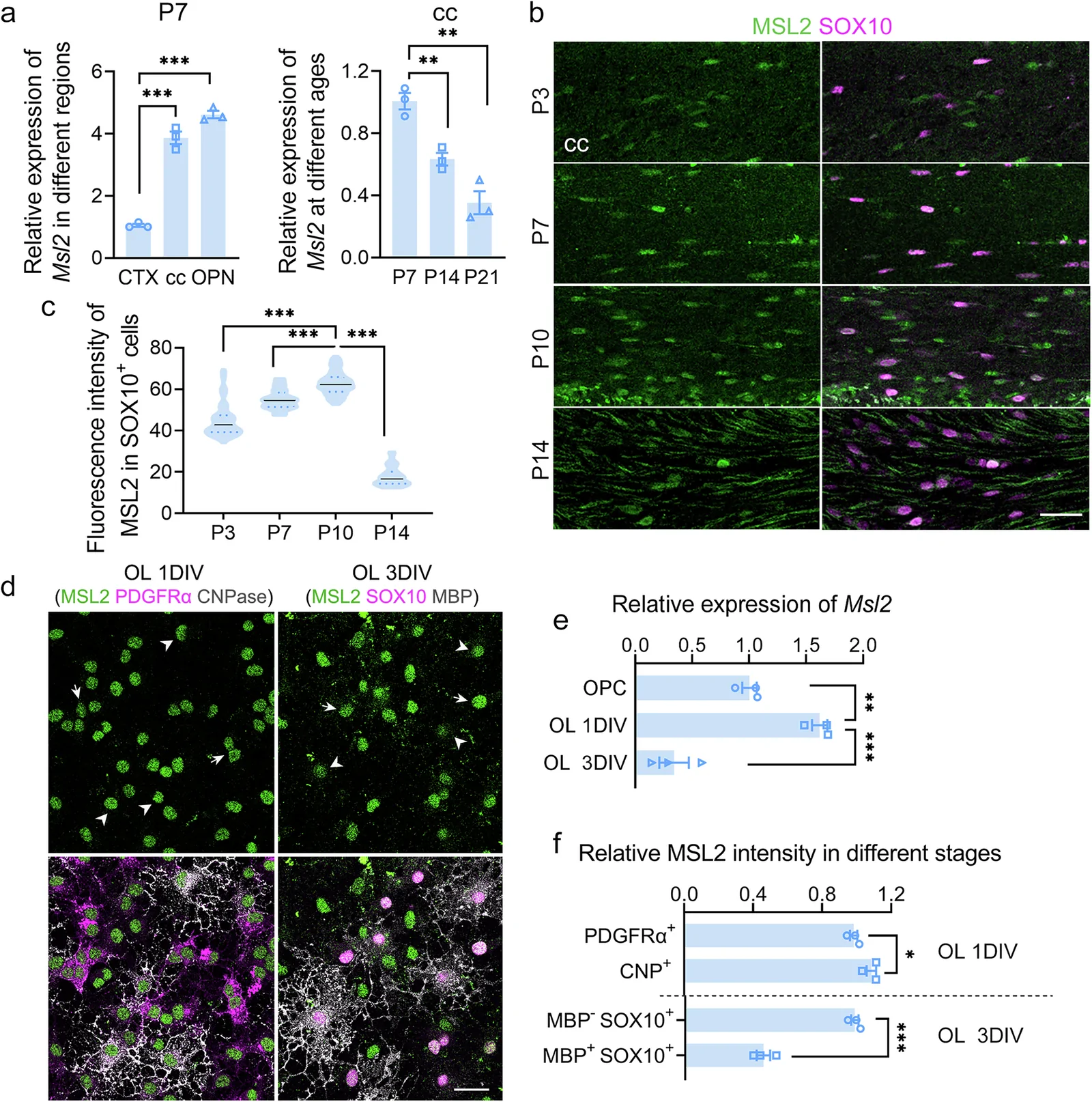
Stage-dependent expression pattern of MSL2 during OPC differentiation. **a** Relative expression of *Msl2* in different brain regions from P7 mice (left panel) and in the corpus callosum (cc) from different ages (right panel). Data are mean ± s.e.m. Each data point represents one individual mouse (*n* = 3 mice per group). One-way ANOVA, **P < 0.01, *** *P* < 0.001. **b** Immunofluorescence for MSL2 and SOX10 in the cc from mice at different developmental stages as indicated. Scale bar 100 μm. **c** Fluorescence intensity of MSL2 in SOX10^+^ cells at different developmental stages. Data are mean ± s.e.m. (*n* = 120 cells from three mice). One-way ANOVA, ***P < 0.001. **d** Representative images of MSL2 immunostaining during OPC differentiation in vitro. Arrows indicate PDGFRα^+^ cells in OL 1 DIV cultures and MBP^−^ cells in OL 3 DIV cultures; arrowheads indicate CNPase^+^ cells and MBP^+^ cells, respectively. Scale bar 20 μm. **e** Relative expression of *Msl2* in purified OPC, OL 1 DIV, and OL 3 DIV cultures. Data are mean ± s.e.m. Each data point represents one individual mouse (*n* = 3 biologically independent samples per group). One-Way ANOVA, **P < 0.01, ***P < 0.001. **f** Relative fluorescence intensity of MSL2 in different cell stages. Data are mean ± s.e.m. Each data point represents one independent primary cell culture (*n* = 3 independent cultures per group). Two-tailed unpaired *t*-test, *P < 0.05, ***P < 0.001. *CTX* cortex, *OPN* optic nerve.

To examine MSL2 expression in the brain, RNA samples from the gray matter (cortex) and white matter (corpus callosum and optic nerve) of postnatal day 7 (P7) mice were subjected to a quantitative real-time PCR (qRT-PCR) assay. We found that the white matter samples exhibited much higher *Msl2* levels than those in the cortex (Fig. 2a), indicating region-specific expression of MSL2 in the brain at this stage. In addition, we compared *Msl2* mRNA levels in white matter during development. Corpus callosum from P7 mice showed higher *Msl2* expression than P14 and P21 mice (Fig. 2a), suggesting that this gene functions mainly in the early brain developmental stage. Similar results were observed in the immunostaining assay with OL lineage marker during the first two postnatal weeks (Fig. 2b,c). The level of MSL2 immunostaining intensity in SOX10^+^ OL lineage cells reached the highest in P10 corpus callosum and significantly decreased in P14 samples. Furthermore, in purified OL cultures, qRT-PCR and immunostaining assay revealed that the level of MSL2 was the highest in CNPase^+^ newly formed OLs from 1-day in vitro (DIV) OL culture, and significantly decreased in MBP^+^ mature OLs from 3 DIV OL culture (Fig. 2d–f). These results imply the involvement of MSL2 in active myelination.

**Fig. 3:**
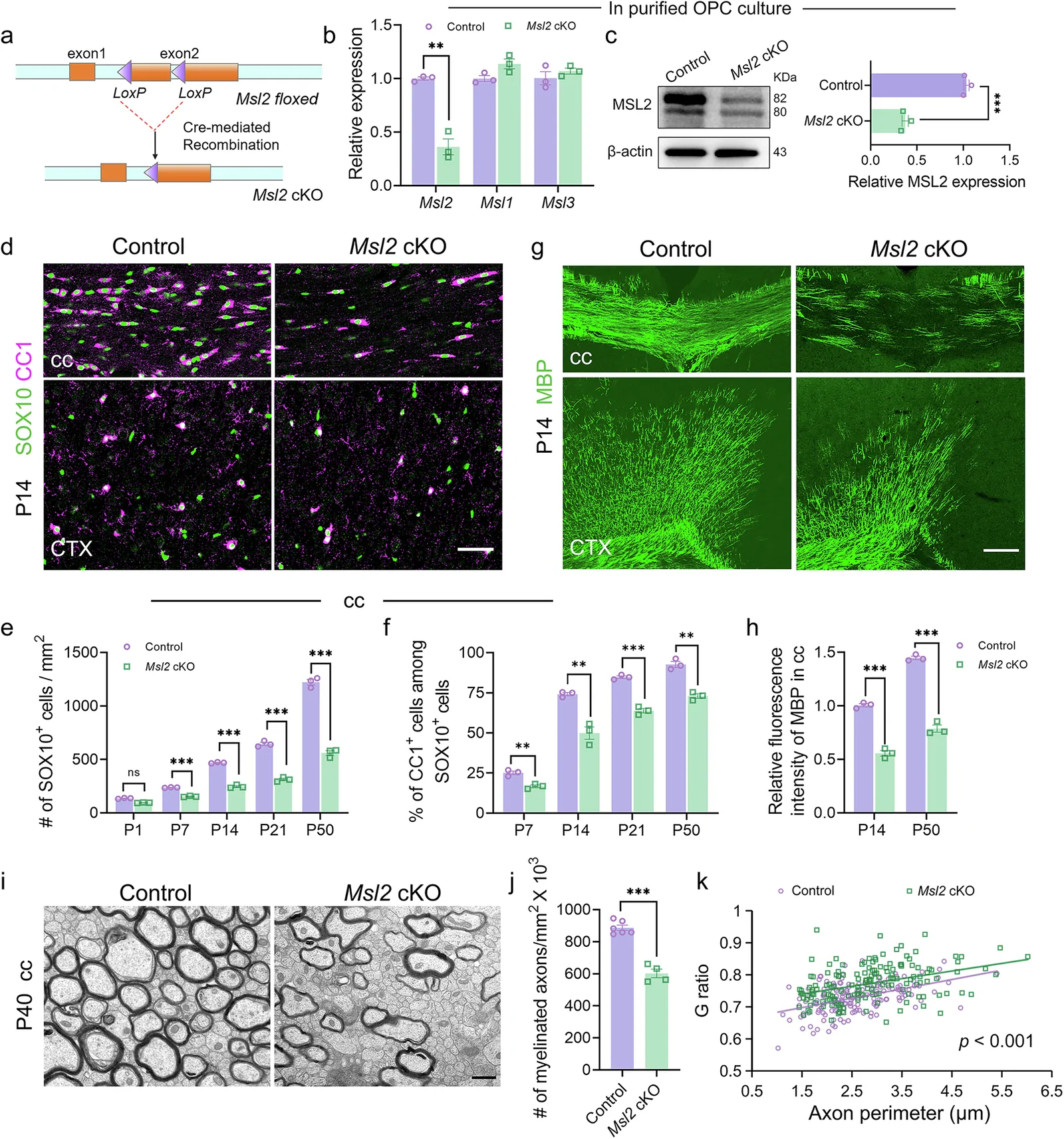
MSL2 is required for OPC differentiation and myelination. **a** A schematic diagram of Cre-mediated excision of floxed *Msl2* exon. **b** Relative expression of *Msl1, Msl2*, and *Msl3* in purified OPC cultures from control and *Msl2* cKO mice. **c** Western blot analysis and quantification of MSL2 levels in OPC cultures from control and *Msl2* cKO mice. **d** Representative immunostaining for CC1 and SOX10 in the cc and CTX from P14 control and *Msl2* cKO mice. Scale bar 100 μm. **e** Quantification of the density of SOX10^+^ cells in cc from control and *Msl2* cKO mice at different ages. **f** Quantification of the proportion of CC1^+^ mature OLs among SOX10^+^ cells in cc from control and *Msl2* cKO mice at different ages. **g** Representative immunostaining for MBP in cc and CTX from P14 control and *Msl2* cKO mice. Scale bar 200 μm. **h** Quantification of the relative intensity of MBP in cc from control and *Msl2* cKO mice at P14 and P50. **i** Representative electron micrographs of cc from P40 control and *Msl2* cKO mice. Scale bar 0.5 μm. **j** Quantification of the density of myelinated axons in the defined areas from cc in P40 control and *Msl2* cKO mice. **k** The g-ratios vs. axonal perimeters in cc from control and *Msl2* cKO mice. Unpaired *t*-test with Welch’s correction, *P* < 0.001 (control, *n* = 160; *Msl2* cKO, *n* = 158 myelinated axons counted from three animals each genotype). Data are mean ± s.e.m. Each data point represents one individual mouse (*n* = 3 mice per group) in b, c, e, f, h, j; or *n* = 6 slides in control and *n* = 4 slides in cKO from three animals per group in j). Two-tailed unpaired *t*-test, n.s. (not significant) *P* > 0.05, **P < 0.01, ***P < 0.001.

According to a recent study, de novo truncating variants of *MSL2* lead to lower levels of intact MSL2 in patients with NDDs^6^. To reveal the function of MSL2 in OPC development, we deleted the exon 2 of *Msl2* from early OL lineage stage by crossing *Msl2*^flox/flox^ mice with the *Olig1-*Cre line^17^ (Fig. 3a). This exon encodes two evolutionarily conserved domains in MSL2, an N-terminal RING finger domain and a C-terminal CXC domain^26^, and deletion of this region results in a loss-of-function mutation of the *Msl2* gene. *Olig1*Cre^+/−^ (*Msl2*^+/+^ *Olig1*Cre^+/−^) littermates were used as controls. To assess Cre-mediated *Msl2* depletion in OL lineage, we detected *Msl2* levels in purified OPCs from *Msl2* mutants using qRT-PCR and western blot assay, and revealed that the expression of *Msl2* was significantly reduced in the *Msl2* cKO group (Fig. 3b,c). The transcripts of other two MSL family members, *Msl1* and *Msl3*, were unchanged in *Msl2* cKO group (Fig. 3b). Besides, immunostaining for MSL2 in P6 brain slices showed that the percentage of MSL2^+^ cells among SOX10^+^ OLs was greatly reduced after *Msl2* depletion (Supplementary Fig. 2a,b), but the level of MSL2 in NeuN^+^ mature neurons was comparable between the two groups (Supplementary Fig. 2c).

To investigate the effects of MSL2 on OL development in the brain, we examined the number of OL lineage cells and the percentage of mature OLs in the mutant brain. The density of SOX10^+^ OL lineage cells in *Msl2* cKO mice was comparable to that in control mice at P1 (Fig. 3e and Supplementary Fig. 2d), but it was significantly reduced from P7 to P50 in the corpus callosum (white matter) and in the cortex (gray matter) (Fig. 3d,e and Supplementary Fig. 2e,f). Besides, the percentage of CC1^+^ mature OLs (Fig. 3d,f and Supplementary Fig. 2e,g) and the level of myelin basic protein (MBP) or myelin marker were also substantially decreased in the cortex and the corpus callosum of *Msl2* cKO mice at different ages, from P7 to 7 months (Fig. 3g,h and Supplementary Fig. 2h–k). In addition, electron microscopy revealed that the number of myelinated axons was significantly reduced in *Msl2* mutants compared with controls at P40 corpus callosum and optic nerve (Fig. 3i,j and Supplementary Fig. 2l,m). The myelinated axons in *Msl2* cKO mice were characterized by a higher g-ratio and thinner myelin sheaths than those in control mice (Fig. 3k and Supplementary Fig. 2n). These results indicate the stage-dependent effect of MSL2 and suggest that the impaired myelination in *Msl2* cKO mice may result from the downsized pool of OL lineage. We then examined the proliferation and apoptosis of OLs.

**Fig. 4:**
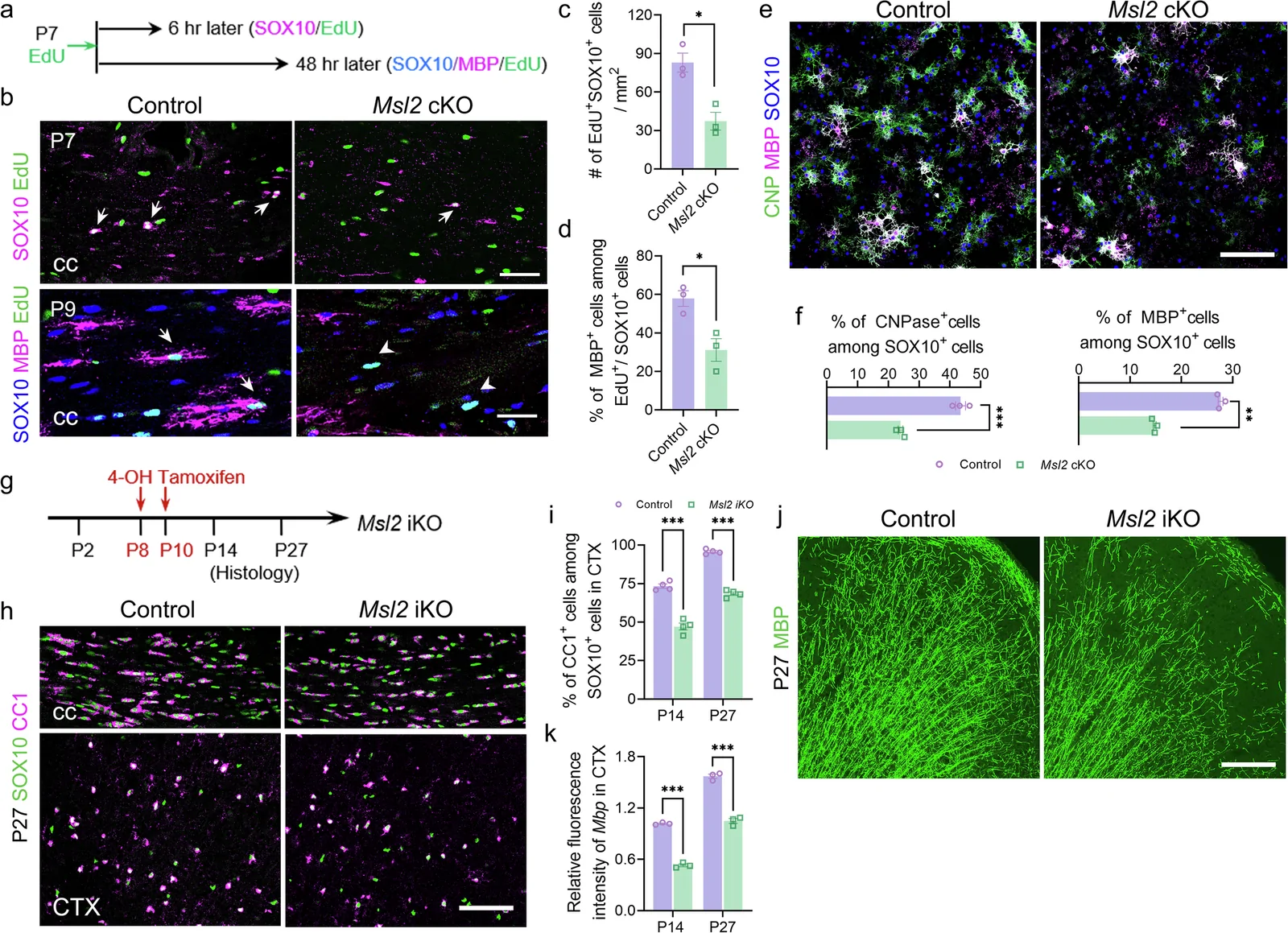
Cell-autonomous role of MSL2 in OPC differentiation. **a** A diagram showing EdU incorporation assay in P7 mice. **b** Immunostaining of SOX10 with EdU to identify proliferating OPCs, or SOX10, MBP, and EdU to identify newly generated OLs in cc from P7 or P9 control and *Msl2* cKO mice. Arrows indicate SOX10/EdU double-positive cells in P7 samples, or MBP/EdU/SOX10⁺ cells in P9 control. Arrowheads indicate SOX10/EdU⁺ cells negative for MBP in P9 *Msl2* cKO group. Scale bar 100 μm. **c** Quantification of the density of EdU/SOX10 double-positive cells. **d** Quantification of the percentage of MBP⁺ cells among EdU/SOX10 double-positive cells in cc from P9 control and *Msl2* cKO mice. **e** Representative immunostaining of CNPase, MBP, and SOX10 in OL 2 DIV cultures from control and *Msl2* cKO mice. Scale bar 50 μm. **f** Quantification of the percentage of CNPase⁺ and MBP⁺ cells among SOX10⁺ cells in OL cultures, respectively. **g** A diagram showing 4-OH tamoxifen administration to induce the Cre recombination in *Msl2* OPC-iKO (*Msl2*^flox/flox^; NG2-CreER) mice. **h** Representative immunostaining of CC1 and SOX10 in cc and CTX from P27 control and iKO mice. Scale bar 100 μm. **i** Quantification of the percentage of CC1⁺ cells among SOX10⁺ cells in cc from P14 and P27 control and iKO mice. **j** Representative immunostaining of MBP in CTX from P27 control and iKO mice. Scale bar 100 μm. **k** Quantification of the intensity of MBP staining in CTX from control and iKO mice at P14 and P27. Data are mean ± s.e.m. Each data point represents one individual mouse (*n* = 3 or 4 animals per group, or *n* = 3 independent cultures per group). Two-tailed unpaired *t*-test; *P < 0.05, **P < 0.01, ***P < 0.001.

The TUNEL assay for P6 mice brain sections did not reveal increased apoptotic cells in *Msl2* mutants (Supplementary Fig. 3a,b). However, the EdU incorporation assay in P7 mice identified fewer proliferative OPCs after *Msl2* loss (Fig. 4a–c). Moreover, most of these EdU-incorporated OPCs developed into newly generated MBP^+^ OLs at P9 in control mice, whereas fewer EdU/MBP double-positive cells were observed in *Msl2* cKO mice (Fig. 4b,d), indicating impaired differentiation after *Msl2* ablation.

To further dissect MSL2 function in OL differentiation independent of cell cycle effects, we used purified differentiating OL cultures from *Msl2* cKO mice and incorporated the NG2-Cre^ER^ mouse line into our analyses. Primary OPCs from the neonatal cortices of control and *Msl2* cKO pups were treated with T3 to induce differentiation immediately after plating, and immunostaining showed that the percentage of CNPase^+^ and MBP^+^ cells among SOX10^+^ cells in *Msl2*-deficient cultures was significantly decreased compared with the control group at 2 DIV (Fig. 4e,f), suggesting that *Msl2*-depleted OPCs show intrinsically reduced differentiation capacity.

Moreover, we bred *Msl2*^fl/fl^ mice with NG2-Cre^ER^ line^18^, an OPC-specific inducible Cre line to generate *Msl2*^fl/fl^; NG2-Cre^ER^ (*Msl2* iKO) mice. To induce Cre recombinase expression, 4-OHT was intraperitoneally injected twice into *Msl2* iKO pups at P8 and P10 (Fig. 4g), triggering *Msl2* deletion at the initiation of OPC differentiation rather than at an earlier proliferative stage (e.g., P2). To validate the appropriateness of this time point, we performed an EdU incorporation assay in wild-type mice at P8, which revealed that nearly 80% of SOX10^+^ cells were NG2^+^ in the corpus callosum, and only 5.48% of NG2^+^ cells were EdU^+^ (Supplementary Fig. 3c,d). These data demonstrate that *Msl2* deletion at this stage primarily affects OL differentiation rather than proliferation or cell cycle progression. NG2-Cre^ER^ mice injected with 4-OHT served as controls. In P14 and P27 corpus callosum and cortex, we did not detect obvious changes in the density of SOX10^+^ cells in *Msl2* iKO mice (Fig. 4h and Supplementary Fig. 3e,f). However, a reduction in the percentage of CC1^+^ mature OLs and MBP intensity was revealed in *Msl2* iKO mice (Fig. 4h–k, Supplementary Fig. 3g,h). Together, these results indicated that MSL2 is essential for both proper cell division and the initiation of OPC differentiation.

### *Msl2* cKO mice presented NDD behaviors

**Fig. 5:**
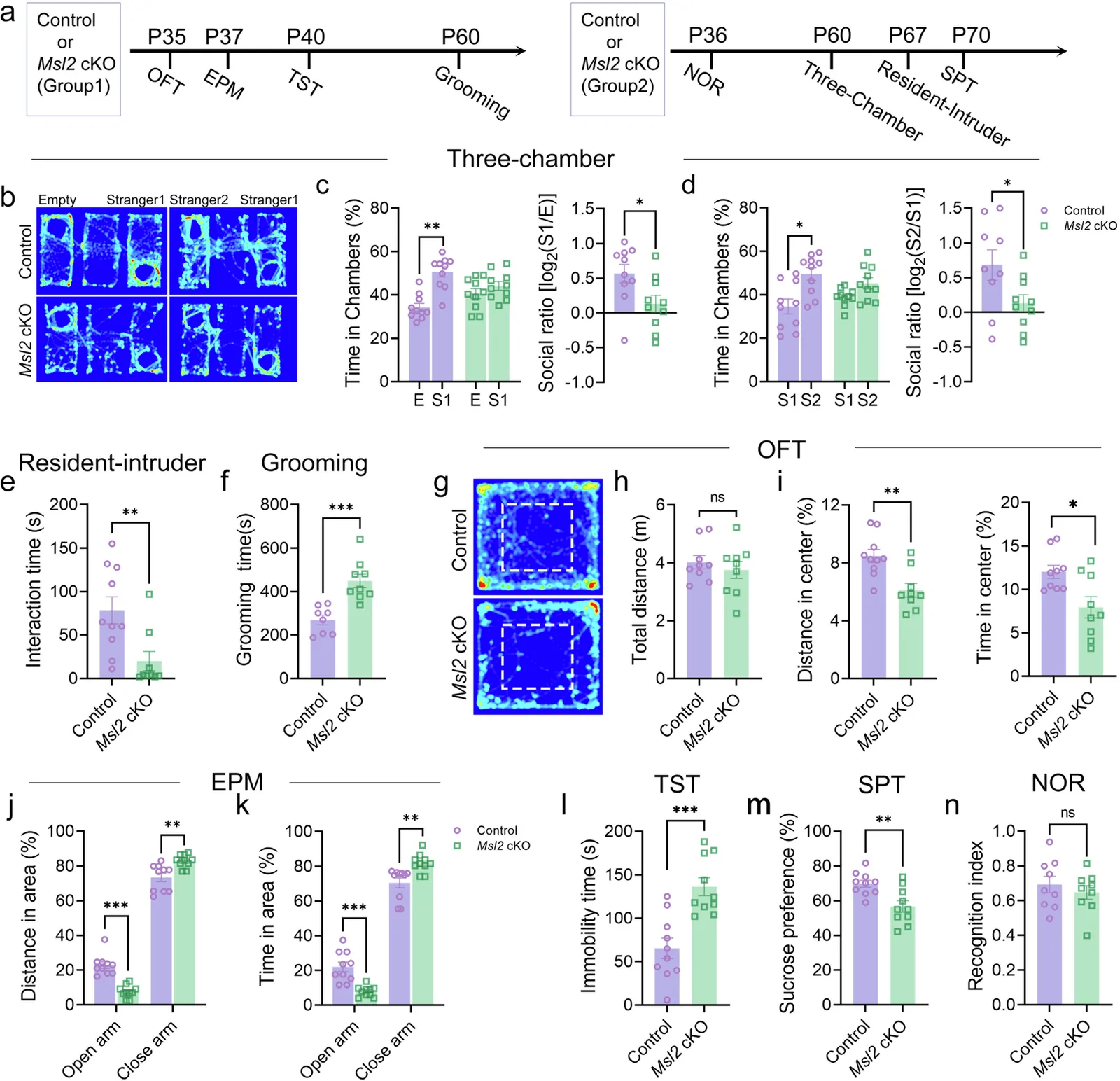
*Msl2* loss in OLs leads to social withdrawal and anxious–depressive behaviors in the mice. **a** A schematic diagram for the mouse behavioral test plan. **b** Representative heat maps of mice movements in the three-chamber test. **c** Percentage of time spent in different chambers and logarithm of social ratio during the first stage. **d** Percentage of time spent in different chambers and logarithm of social ratio during the second stage. **e** Comparison of the interaction time of resident control or *Msl2* cKO mice with intruder mice. **f** Comparison of the spontaneous grooming time of control and *Msl2* cKO mice. **g** Representative heat maps of free movements of mice in an open field test (OFT). **h** Comparison of the total traveling distances between control and *Msl2* cKO mice in a 10-minute test. **i**, Percentage of traveling distance and time for control and *Msl2* cKO mice in the center zone of open field. **j**, **k** Percentage of traveling distance (**j**) and time (**k**) for control and *Msl2* cKO mice in the indicated arms of elevated plus maze (EPM). **l** Immobility time for control and *Msl2* cKO mice in the tail suspension test (TST). **m** Percentage of sucrose consumption volume for control and *Msl2* cKO mice in the sucrose preference test (SPT). **n** Recognition index for control and *Msl2* cKO mice in the novel object recognition (NOR) test. Data are mean ± s.e.m. Each data point represents one individual mouse (*n* = 8–10 mice in each group). Two-tailed unpaired *t*-test in **c**, **d**, **f**, **h**, **i**, **l**, **m** and **n**; two-tailed unpaired *t*-test and Mann–Whitney test in **e**, **j** and **k**. *n.s.* not significant, *P* > 0.05, *P < 0.05, **P < 0.01, ***P < 0.001.

To test the functional consequences of *Msl2* ablation in OLs, a series of behavioral tests was conducted to examine how these changes affected animal behaviors (Fig. 5a). We first analyzed social interaction in these mice. In a three-chamber sociability test, *Msl2* cKO mice showed no preference for the stranger mouse (S1) over the empty box (E) in the first phase of the test, unlike control mice (Fig. 5b,c). In the subsequent social novelty phase, the mutant failed to interact with the second stranger mouse (S2) (Fig. 5b,d), indicating deficits in both social preference and social novelty recognition. Moreover, in a resident–intruder test, *Msl2* cKO mice seldom interacted with intruder mice and showed strong social avoidance toward other mice (Fig. 5e). The self-grooming test indicated repetitive behaviors in the mutant (Fig. 5f).

As comorbid mood disorders, especially anxiety and depression, in children and adolescents with NDDs have been widely reported^27,28^, we also included tests for these mental conditions. In the open field test, mutant and control mice showed comparable locomotor activity during a 5-minute test (Fig. 5g,h). However, the mutant mice spent less time and traveled a shorter distance in the central area than control mice (Fig. 5i), indicating their increased level of anxiety. In another anxiety test in rodents, the elevated plus maze, *Msl2* cKO mice traveled a shorter distance and spent less time in the open arm than their control littermates at P37 (Fig. 5j,k). These data indicated that OL dysfunction after *Msl2* ablation may cause anxious behaviors in the animals. Besides, as shown in Fig. 5l, the immobility time of P40 *Msl2* cKO mice in the tail suspension test was significantly increased compared with the control mice. Moreover, these mice at P70 showed less sucrose consumption than controls in the sucrose preference test (Fig. 5m).

Finally, in the novel object recognition test, both control and mutant mice showed preference for the novel objects, and there was no significant difference between their recognition index (Fig. 5n). Together, our results indicate that mice developed social interaction withdrawal, repetitive behavior, and anxious–depressive mood after *Msl2* ablation in OLs, with normal cognitive ability.

### *Msl2* ablation disturbed the formation of the mitotic spindle in OPCs

**Fig. 6:**
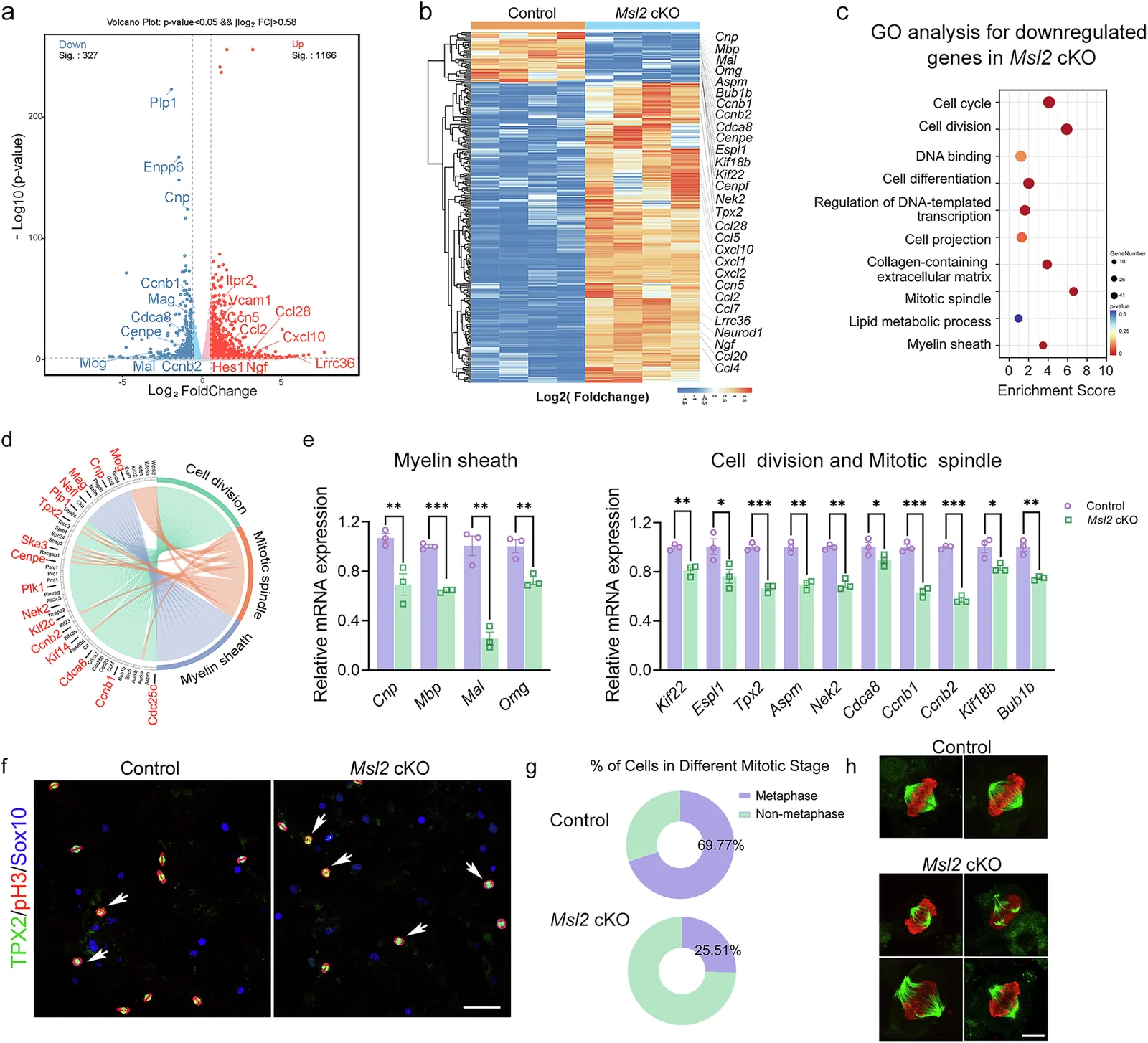
*Msl2* ablation changes transcriptome profiling in OPCs and impairs spindle formation. **a** Volcano plot of genes with |log_2_ fold change| (> 0.58) on X-axis and log_10_ of *P-*value on Y-axis. Samples are purified OPC cultures from control and *Msl2* cKO mice, and each point represents an individual gene. **b** Hierarchical clustering of upregulated and downregulated genes in OPCs from cKO mice (*n* = 4 independent cell cultures per group). **c** Bubble plots of GO analysis for downregulated genes in *Msl2* cKO mice. **d** Circos diagram of representative GO terms, cell division, mitotic spindle, and myelin sheath, for downregulated genes in *Msl2* cKO mice. Representative genes were labeled red. **e** The qRT-PCR validation of representative downregulated genes in *Msl2* cKO OPCs. Data are mean ± s.e.m. of transcript levels relative to control after normalization (*n* = 3 biologically independent experiments each performed in technical triplicate). Two-tailed unpaired *t*-test: *P < 0.05, **P < 0.01, ***P < 0.001. **f** Representative immunostaining of TPX2, pH3, and Sox10 for metaphase synchronized OPC cultures from control and *Msl2* cKO mice. Arrows indicate cells in non-metaphase, such as prophase or prometaphase. Scale bar 50 μm. **g** Quantification of the percentage of pH3^+^ cells in metaphase and non-metaphase in both control and *Msl2* cKO groups. **h** Representative spindle shapes in control and *Msl2* cKO OPCs. Scale bar 10 μm.

To further identify the downstream targets of MSL2 in OLs, we compared the transcriptome profiles of purified OPC cultures from control and *Msl2* cKO mice. There are 1,166 genes upregulated and 327 genes downregulated in OPCs from the *Msl2* cKO mice group (Fig. 6a,b). GO analysis for these downregulated transcripts revealed terms associated with cell cycle, cell division, differentiation, and myelin sheath (Fig. 6c,d), which are consistent with the interrupted cell proliferation and hypomyelination phenotype in the mutant. The qRT-PCR assay confirmed the decreased expression of several representative genes involved in cell division, mitotic spindle, and myelin sheath after *Msl2* ablation (Fig. 6e).

Among these genes, *Aspm* (assembly factor for spindle microtubules) and *Tpx2* (TPX2 microtubule nucleation factor) are essential for spindle architecture^29,30^; *Bub1b* (BUB1 mitotic checkpoint serine/threonine kinase B) encodes a kinase involved in spindle-assembly checkpoint function^31^. Therefore, we determined the biological significance of these downregulated genes in mitotic progression after *Msl2* depletion. OPC cultures were synchronized in prometaphase using thymidine, followed by the Eg5 inhibitor STLC, and were subsequently released into culture medium containing MG132 to allow full establishment of bipolar spindles^32^ (Supplementary Fig. 4a,b). We observed that 69.77% of cells labeled with the mitotic marker pH3 were arrested in metaphase in the control group, but only 25.51% of pH3^+^ cells were in metaphase in the mutant group (Fig. 6f,g). Most pH3^+^ cells in cKO cultures were in prophase or prometaphase, thereby delaying spindle establishment. In addition, the spindle shapes in metaphase showed notable abnormalities after *Msl2* ablation, including loose chromosome compaction, triple- or multipolar spindle, and twisted spindle (Fig. 6h). These defects may account for the prolonged mitotic phase and result in a reduced number of OL lineage cells in *Msl2* cKO brain.

For these upregulated genes, we found that gene terms, such as inflammatory response, innate immune response, and cytokine activity, were highly enriched in *Msl2* cKO OPCs (Supplementary Fig. 5a,b). Among these genes, several chemokines and cytokines, such as *Cxcl1, Cxcl2, Ccl4,*and *Ccl20*, showed higher expression in *Msl2*-ablated OPCs (Supplementary Fig. 5c). This observation suggests that depletion of MSL2 may induce immunomodulatory properties in OPCs, potentially enabling them to actively orchestrate the immune-inflammatory response in mutant mice.

### MSL2-mediated histone methylation is essential for the proper development of OPCs

**Fig. 7:**
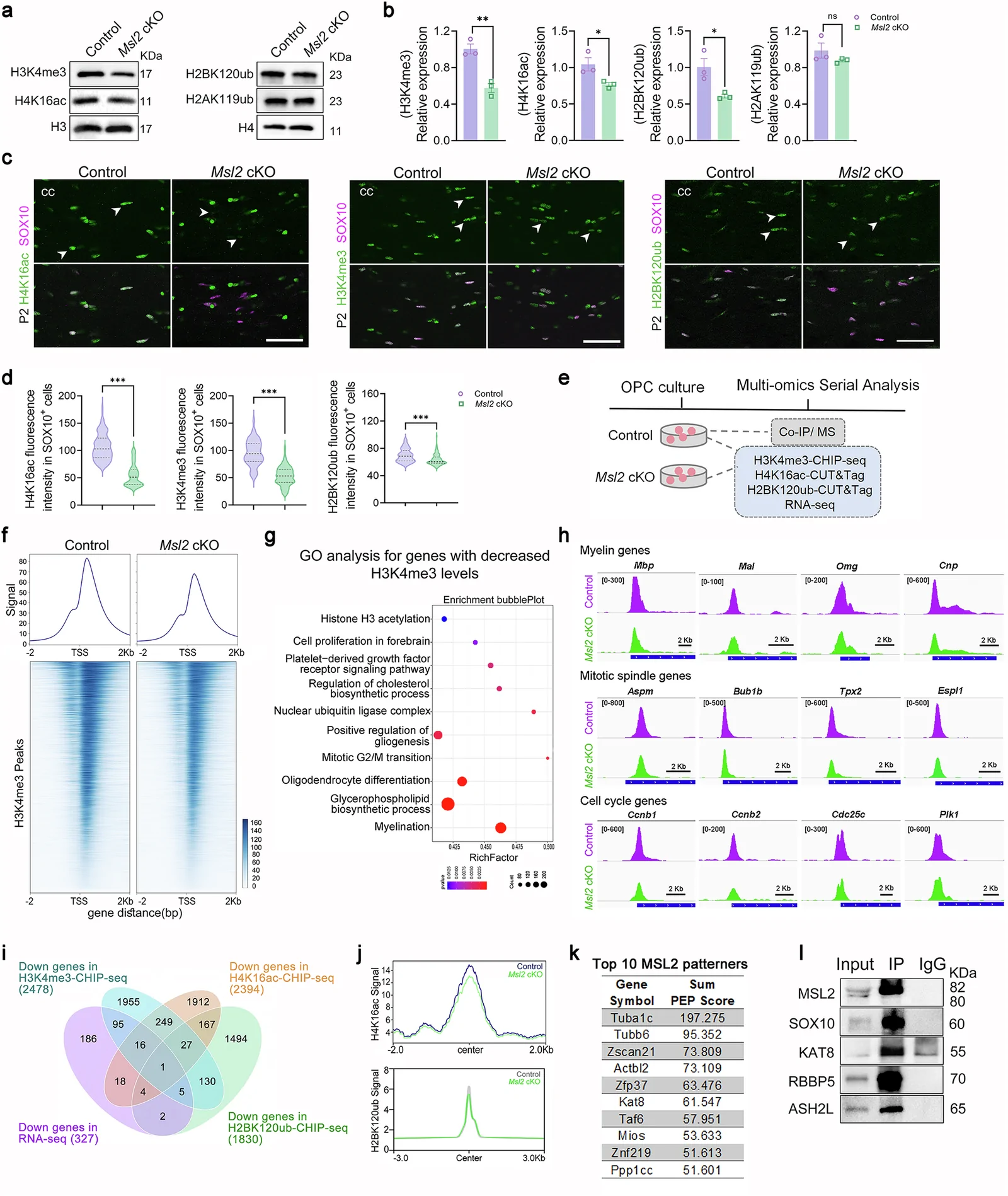
MSL2-mediated histone methylation is essential for the proper development of OPCs. **a** Western blot analysis for H3K4me3, H4K16ac, H2BK120ub, and H2AK119ub in OPCs from control and *Msl2* cKO mice. H3 and H4 were used as loading controls. **b** Relative levels of H3K4me3, H4K16ac, H2BK120ub, and H2AK119ub in control and cKO OPCs. Data are mean ± s.e.m. Each data point represents one independent primary cell culture (*n* = 3 independent cultures per group). Two-tailed unpaired *t*-test, n.s. (not significant), *P* > 0.05, *P < 0.05; **P < 0.01. **c** Immunostaining of H4K16ac, H3K4me3, and H2BK120ub in P2 *Msl2* cKO mice. Scale bar 50 μm. **d** Quantification of the immunofluorescence intensity of the indicated histone modification in SOX10^+^ cells. Data are mean ± s.e.m. (*n* = 100–150 cells from three mice). Two-tailed unpaired *t*-test, ***P < 0.001. **e** Schematic strategy for analyzing MSL2-involved gene expression in OPCs. **f** Metaplots and heat maps for H3K4me3 ChIP-seq peaks from control and *Msl2* cKO OPCs, showing ± 2 kb around TSS. **g** GO analysis for genes with decreased H3K4me3 levels in *Msl2* cKO OPCs. **h** Snapshots of H3K4me3 profiles for myelination genes, mitotic spindle genes, and cell proliferation genes in control and *Msl2* cKO OPCs. Tracks were shown in IGV 2.4.8. **i** Venn diagram of downregulated genes with reduced H3K4me3, H4K16ac, and H2BK120ub levels. **j** The average distribution of H4K16ac and H2BK120ub signals in control and *Msl2* cKO OPCs. **k** Gene list of top ten MSL2 binding partners identified by co-immunoprecipitation coupled with mass spectrometry (Co-IP/MS). **l** Western blot analysis for KAT8, RBBP5, ASH2L, and SOX10 in MSL2 immunoprecipitated products from OPCs.

As mentioned above, MSL2 may indirectly induce H3K4 and H3K79 methylation across the genome through the addition of ubiquitin to H2BK34 and H2BK120^33^. As one of the components in the MSL–MOF complex, MSL2 also participates in the acetylation of H4K16. To elucidate the underlying epigenetic mechanism of MSL2-mediated regulation in OLs, we first examined the global level of H2BK120ub, H3K4me3, and H4K16ac in OPC cultures. Western blot assay revealed that *Msl2* deletion reduced the levels of all three types of modifications at varying degrees, but did not affect the levels of other unrelated histone modifications, such as H2AK119ub (Fig. 7a,b). Similar results were observed in P2 *Msl2* cKO mice (Fig. 7c,d) and P8 *Msl2* iKO mice (Supplementary Fig. 6), which showed specific reduction of H2BK120ub, H3K4me3, and H4K16ac fluorescence intensity in SOX10^+^ cells after *Msl2* ablation.

To investigate the changes of three types of histone modification, we performed ChIP-Seq or CUT&Tag in OPC cultures from control and *Msl2* cKO mice (Fig. 7e). As one of the products from MSL2-mediated '*trans*-tail regulation', H3K4me3 has been considered as an active transcription and open chromosome marker, which is essential for transcriptional initiation^34^. The genomic locus distribution of H3K4me3 was comparable between groups, with 14.42% and 14.97% peaks in promoter regions from control and *Msl2* cKO group, respectively (Supplementary Fig. 7a). However, the depletion of *Msl2* induced a dramatic reduction of H3K4me3 peak signals (Supplementary Fig. 7b), especially near the TSS (Fig. 7f), which indicates the essential role of MSL2 in maintaining dynamic modification of H3K4me3 in OLs. Further GO analysis indicated that gene terms of myelination and OL differentiation were lost in *Msl2* cKO OPCs. In addition, epigenetic modification-related gene terms and gene terms involving cell proliferation and mitotic G2/M transition were also enriched in these downregulated genes (Fig. 7g). The integrative genomics viewer (IGV) images for H3K4me3 peaks illustrated representative genes with lower peaks near their TSS in *Msl2* cKO OPCs (Fig. 7h). Other genes with consistent level of H3K4me3 after *Msl2* ablation were shown in Supplementary Fig. 7c. Notably, the crosslink analysis for H3K4me3 modification and RNA sequencing data revealed that 177 genes with lower H3K4me3 level were downregulated in *Msl2* cKO OPCs, accounting for 35.78% of total downregulated genes (Fig. 7i).

For H4K16ac identified in CUT&Tag, most peaks were located in intergenic regions of annotated RefSeq genes in both groups (Supplementary Fig. 8a). Global H4K16ac levels were slightly reduced in *Msl2-*deficient OPCs (Fig. 7j and Supplementary Fig. 8b), consistent with previous reports in mouse embryonic stem cells, neural progenitors, and human THP-1 cells^10,11,35^. This observation implies that MSL2 depletion does not compromise the protein stability of other MSL complex components in OPCs. GO analysis revealed that genes with diminished H4K16ac peaks in *Msl2* cKO OPCs were highly enriched for terms related to cell projection organization, lipid and sphingolipid metabolism and localization, and cell morphogenesis (Supplementary Fig. 8c). Integrative analysis of H4K16ac CUT&Tag and RNA sequencing data further showed that only 39 out of 327 downregulated genes (11.92%) exhibited concordant reductions in H4K16ac after *Msl2* ablation (Fig. 7j). Regarding H2BK120ub, an indirect target of MSL2 E3 ubiquitin ligase activity, we detected a mild decrease in *Msl2* cKO OPCs (Fig. 7j and Supplementary Fig. 8d–f). Overlap analysis demonstrated that 8.9% of genes with reduced H2BK120ub also displayed lower H3K4me3 signals upon MSL2 loss, and only 12 out of 1,830 genes with diminished H2BK120ub were transcriptionally downregulated. Together, these data suggest that H2BK120ub is not a major driver of H3K4 methylation in OL lineage cells. Collectively, our findings indicate that MSL2 depletion does not substantially alter MOF catalytic activity, and that its indirect ubiquitin ligase target H2BK120ub is not essential for global transcriptional regulation in OLs. Instead, MSL2 likely modulates transcriptional programs in these cells primarily through H3K4me3 modifications.

Finally, to explore MSL2 binding partners in OLs, we performed a proteomic study using co-immunoprecipitation of the protein extractions from OPCs. By comparing with IgG pull-down results, 160 proteins were identified as MSL2 interactors (Supplementary Table 3), and the top 10 patterners are listed in Fig. 7k. Among these genes, we found a well-known functional partner of MSL2, histone acetyltransferase KAT8 or MOF, and we validated its appearance in MSL2-IP products in the western blot assay (Fig. 7l). Notably, ASH2L and RBBP5, the core subunits of the COMPASS-like H3K4 methyltransferase complex, were also identified as MSL2-interacting proteins (Supplementary Table 3). This interaction was further validated in western blot assays on MSL2-IP eluates (Fig. 7l). In addition, one OL lineage-specific transcription factor, SOX10, appeared as an MSL2 binding partner (Fig. 7l and Supplementary Table 3), indicating its role as a leading protein to guide the MSL complex to its target genes in OLs.

Overall, we revealed the crucial function of a newly identified causal gene for NDDs, MSL2, in modulating OL differentiation. By investigating the cellular context-dependent genome-wide distribution of two major MSL2-mediated histone modifications, we propose that MSL2 exhibits unique targeting specificity in OLs that ensures the timely maturation of these cells during development. These findings point to the potential benefit of modulating MSL2 complex activity to enhance (re)myelination programs in neurological disorders such as ASD or after demyelination.

## Discussion

Converging clinical evidence indicates a strong association between *MSL2* dysfunction and white matter–OL development. In the largest cohorts reported to date^6,7^, nearly half of patients with pathogenic *MSL2* variants demonstrated brain MRI abnormalities—most commonly delayed myelination and white matter volume loss, occasionally accompanied by corpus callosum hypoplasia or ventriculomegaly. In line with these findings, the three individuals we report presented with neurodevelopmental impairment characterized by language deficits, motor delay, intellectual disability, and autistic features, with neuroimaging further supporting the link between *MSL2* variants and white matter pathology. Collectively, these data underscore white matter vulnerability as a recurrent hallmark of MSL2-related NDDs.

Our study identifies MSL2 as a critical epigenetic modulator of OL lineage progression, with its expression peaking during early postnatal myelination. The severe hypomyelination observed in *Msl2* cKO mice aligns with clinical reports of white matter defects in patients with NDDs carrying *MSL2* variants^5–7^. Notably, the stage-dependent effects of *Msl2* ablation suggest that MSL2 primarily governs the transition from proliferative OPCs to differentiating OLs, rather than lineage commitment. The finding that *Msl2* cKO uniquely combines cell cycle dysregulation (EdU^+^ reduction and less metaphase proportions) with abnormal mitotic spindle formation further implicates its role in expanding the OPC pool during the early postnatal stage; this result is highly consistent with the transcription profile after *Msl2* ablation. As one of the MSL2 targets in OPCs, ASPM is a microcephaly-associated protein family and its knockout showed spindle defects, such as a strong increase in multipolar spindles and spindle misorientation^29,36^. TPX2 is a microtubule-associated protein that is required for stimulating microtubule assembly during mitotic spindle formation. Animal studies showed that TPX2 null embryos arrest at the morula stage with defective mitotic spindles^37^. The BUB1B protein localizes to the kinetochore and plays a role in the inhibition of the anaphase-promoting complex/cyclosome (APC/C), delaying the onset of anaphase and ensuring proper chromosome segregation^38,39^. We speculate that these downregulated genes act together to disturb mitotic progression and reduce the population of OL lineage cells in *Msl2* cKO mice. Together with the pathological changes in the cytoarchitecture of myelin in the *Msl2* mutant brain, the final delayed myelination rate in both white and gray matter may result in dysfunctional connectivity in critical pathways, causing abnormal behaviors in these mice. We believe that further examination of critical neural circuit alterations associated with hypomyelination in *Msl2* mutant brain will provide more detailed explanations for the anatomical and functional involvement of MSL2 in inducing abnormal behaviors.

According to the Diagnostic and Statistical Manual of Mental Disorders (DSM-5), ASD comprises two symptomatic domains: deficits in communication and social interaction, and repetitive and restricted behavior^40^. Notably, individuals with ASD are thought to be at a disproportionate risk of developing mental health comorbidities, with anxiety and depression being considered most prominent amongst these^27,41,42^. The *Msl2* cKO mouse recapitulates the core behavioral triad of ASD: social interaction deficits, repetitive grooming, and comorbid anxiety–depression. These features make the mice a potential animal model to study the contribution of epigenetically regulated myelin function in ASD.

As mentioned above, the behavior phenotypes may arise from disrupted connectivity due to hypomyelination, as evidenced by reduced corpus callosal integrity and prior ASD imaging studies^2,43^. Furthermore, the increased transcript level of several cytokines in OPCs from *Msl2* cKO mice introduces an alternative mechanism, neuroinflammation, potentially exacerbating behavioral deficits. This aligns with a recent work linking OL-derived cytokines (e.g., *Cxcl1*, *Ccl4*) to microglial priming in NDDs^44^. Together, these studies provide further directions for investigating the role and mechanism of MSL2 in cell–cell interactions and neuroinflammation in ASD.

Chromatin modifications create a complex epigenetic landscape whose regulation in both time and space is thought to be critical for the establishment and maintenance of OL identity. As a dual player, MSL2 is implicated in histone modifications through the ubiquitination of histone H2B^8^ and acetylation of histone H4 on K16^14,15^. H2BK34ub is the direct product of MSL2, but it shows low abundance in HeLa cells^8^; another indirect ubiquitination site is H2BK120, which is catalyzed by RNF20/40 through MSL2-facilitated chromatin association^45^. Notably, H2B ubiquitination can further stimulate histone methyltransferases SET1 or DOT1L, which mediate histone H3 lysine 4 (H3K4) and lysine 79 (H3K79) methylation, respectively^3,46^. These histone modifications are all linked to active transcription and may influence transcription initiation, transcription elongation, and genomic stability^10,47–49^.

As one of the intermediates in '*trans*-tail regulation', the H2BK120ub modification is more dynamic^33^. However, the machinery that installs H3 methylation is much more diverse, which makes the product relatively stable. In addition, as a major product catalyzed by the MSL complex, H4K16ac is highly abundant and potentially represents a hallmark of open chromatin and active transcription^10,50^. Our data highlight H3K4me3 as the dominant downstream target of MSL2 in OLs, despite its canonical role in the MSL–MOF complex for H4K16ac^51,52^. This parallels previous findings about H4K16ac changes after MSL2 loss in mouse neural progenitors and patient-derived reprogrammed cells carrying *MSL2* variants^6^. Moreover, the integrative analysis with transcriptomic data demonstrated a markedly higher concordance between decreased H3K4me3 levels and downregulated gene expression. This discrepancy further reinforces the functional preference of MSL2 in shaping the OL episignature. Notably, our mass spectrometry analysis identified ASH2L and RBBP5-core subunits of the COMPASS-like H3K4 methyltransferase complex as MSL2-interacting proteins in primary OPCs. This finding establishes a previously unrecognized mechanism for MSL2-mediated H3K4me3 regulation in OPCs, independent of its E3 ubiquitin ligase activity toward H2BK120ub (CUT&Tag profiling showed minimal overlap between genes with reduced H2BK120ub and those with diminished H3K4me3 after *Msl2* ablation). However, the key binding domains between MSL2 and ASH2L–RBBP5, as well as the specific molecular mechanisms by which this interaction modulates the enzymatic activity of the COMPASS-like complex, remain to be elucidated.

Interestingly, the level of H3K4me3 after *Msl2* ablation did not show a striking reduction, and more than 60% of downregulated genes did not show a significant reduction in their H3K4me3 level. We speculate that MSL2 may influence gene expression through alternative mechanisms. For example, beside H3K4 methylation, H3K79 methylation may also contribute to the expression regulation. Therefore, the observed myelination defects may result from the combined regulation of multiple histone modifications. Besides, Sun et al. hypothesized that MSL2 may prevent DNA methylation in mouse neural progenitor cells, creating a methylation-free environment for the binding of methylation-sensitive transcription factors^6^. We envision that it will be interesting to examine mechanistically whether MSL2 may control these modifications in certain target genes in OLs, and exploring the stage-specificity of MSL2 binding during OL differentiation will help elucidate its role from a more comprehensive perspective.

## Supplementary information

Supplementary Information

Supplementary Material
