# Supplementary material for: MSL2 orchestrates oligodendrocyte precursor cell cycle via H3K4me3 to prevent white matter defects in neurodevelopmental disorders
Source: Exp Mol Med. 2026 Oct 6;58(10):3472–85. doi: 10.1038/s12276-026-01858-1 (PMC13736987; doi:10.1038/s12276-026-01858-1)
Supplement: Supplementary file 1 [file 12276_2026_1858_MOESM1_ESM.pdf]

Supplementary Information for

MSL2 orchestrates oligodendrocyte precursor cell cycle via H3K4me3 to prevent white matter defects in neurodevelopmental disorders

Jie Yuan *et al.*

*Corresponding authors. Email: xianghuizhao@fmmu.edu.cn, shengxi@fmmu.edu.cn, guohui2@csu.edu.cn

This PDF file includes:

Supplementary Table 1 Detailed clinical information of three patients with pathogenic MSL2 variants

Supplementary Table 2 Primer sequences for qRT-PCR assay of target genes

Supplementary Table 3 List of proteins identified by mass spectrometry in the pull-down products of endogenous MSL2

Supplementary Fig.1 Immunostaining of MSL2, H4K16ac and H3K4me3 in immortalized lymphocytes from patient and controls

Supplementary Fig.2 MSL2 is required for OL differentiation and myelination

Supplementary Fig.3 *Msl2* iKO mice recapitulate the hypomyelination phenotype as in *Msl2* cKO mice

Supplementary Fig.4 Assessment of cell synchronization by fluorescence microscopy

Supplementary Fig.5 *Msl2* ablation induces upregulation of several cytokine transcripts in OPCs

Supplementary Fig.6 Immunostaining of H4K16ac, H3K4me3 and H2BK120ub in P8 *Msl2* iKO mice

Supplementary Fig.7 H3K4me3 profiles in *Msl2* ablated OPCs

Supplementary Fig.8 H4K16ac and H2BK120ub profiles in *Msl2* ablated OPCs

Supplementary Materials and Methods

Supplementary References

Supplementary Table 1. Detailed clinical information of three patients with pathogenic MSL2 variants.
Features most closely align with the mouse phenotypes were in red.

Case Index	Case 1	Case 2	Case 3
Region (Cohort)	Switzerland	Australia	China
Country, ethnicity information	Switzerland, Italian origin	Australia	China, Han origin
MSL2 Variant (Isoform: NM_018133.3)	NM_018133.4:c.527_531del, p.(Pro176LeufsTer2)	c.1429C>T; p.(Arg477Ter)	3q22.3(136,176,426-136,259,321)x1(82.896 kb)
Variant Detection Method	WES	WES	WGS
Variant inheritance	*de novo*	*undetermined*	*de novo*
Variant interpretation (ACMG criteria)	P	P	P
Age at diagnosis (y)	17 y	4 yr	3 yr
Age at last examination (y)	18 y	4 yr	6 yr
Gender	female	male	female
Neonatal			
Delivery - weeks gestation	42+0 GA	39	36
Delivery - c-section or vaginal (dystocic delivery?)	vaginal	LSCS	vaginal

Birth weight (w/%)	3500 g (P25-50)	2940 g	2900 g
Birth length (w/%)	51 cm (P25-50)	50	50 cm
Birth OFC (w/%)	35 cm (P25-50)	32	/
Infant feeding difficulties	no	yes	no
Failure to thrive	no	no, drooling	no
Neurodevelopmental			
Autism spectrum disorder (criteria)	no	autistic features (no official diagnosed)	yes
Speech-language problems	yes (first words with 2 y)	yes	yes
Motor delay	yes (free walking with 3 y)	yes (severe)	yes
Intellectual disability	yes (estimate moderate)	yes (severe)	yes (estimate moderate)
Regression of developmental milestones	no	no	no
other developmental problems?	no	dystonia	no
Behavior problem			
Anxiety (type)	no	yes	no
ADHD (criteria)	no	no	no

Repetitive behavior	no	no	no
Obsessive behavior	no	no	no
Aggressive behavior	no	yes	no
Self-injury behavior	no	no	no
other behavior problem	no		no
Neurological problem			
Macrocephaly (OFC w/% or SD)	no	no	no
Microcephaly (OFC w/% or SD)	yes (50.7 cm, <<P1)	yes	no
Brain MRI (description of the details)	On day 9, MRI brain: Sagittal and axial T1; axial DWI, T2, FLAIR sequences. There are periventricular white matter signal foci abnormalities noted on both sides at cerebral hemisphere. This is predominantly parietal and occipital distribution seen best on the T1 weighted sequences, hyperintense. No definite cystic change allowing for motion. There is impression of decreased sulcation in general throughout the cerebral hemispheres on both sides allowing for motion. The	Generalized polymicrogyria with abnormal white matters signals and areas of calcification	Scattered punctate T2/FLAIR hyperintensities within the bilateral frontal white matter, indistinctly marginated; mucosal thickening noted in the right maxillary, left sphenoid and bilateral focal ethmoid sinuses.

	FLAIR sequence is markedly degraded by motion, difficult to exclude potential regions of hyperintensity. There is dilatation of the lateral ventricles on both sides.<br><br>At 7 months: There is extensive symmetrical T2 white matter hyperintensity throughout the cerebral hemispheres with reduced cerebral volume and associated ventricular enlargement. There is abnormal cortical sulcation with widespread bilateral polymicrogyria. There are a number of T1 hyperintense foci with corresponding blooming artefact on the SWI acquisitions throughout the periventricular white matter in keeping with punctate calcifications.		
Brain EEG	not performed	diffuse cerebral dysfunction, no definite epileptiform activity or focal dysfunction	no
Seizure (specify type, relation with fever, frequency etc.)	no	focal tonic clonic	no
Epilepsy (specified in details (e.g. EEG during sleep and awake))	no		no

Sleep disturbances	no	yes	no
Other neurological problem?	no		no
Psychiatric problems			
depression	no		no
bipolar disorder	no		no
schizophrenia	no		no
other psychiatric symptoms	no		no
Systemic			
Weight (w/%)	52.9 kg (P25)	21.1 (97th percentile)	20.3kg（P25）
Height (w/%)	173.5 cm (P9097)	100 (50th percentile)	115.8cm（P10）
OFC (w/%)	50.7 cm (<< P1)	45.8 (<2nd percentile)	53cm
Congenital heart defects	no	no	no
Gastrointestinal disturbance	no		no
skeletal alterations	dolichostenomelia (arm spann 13 cm, arm spann to height ratio 1.0548	hip dysplasia, puffy hands	no
visual impairment	yes (strabism and hyperopia)	difficult to know	no

pen	no	bilateral sensorineural hearing loss	no
Previous genetic and metabolic testing			
Karyotype	normal	normal	normal
Fragile X	not performed	normal	normal
Array-CGH	normal	normal	not performed
Gene panel	normal	not performed	not performed
Exome sequencing (other positive findings besides MSL2	no	no	no
others (e.g. metabolic test)	not performed		not performed
Family History (description)			
Family History (description if yes)	normal		normal
Clinical photos available			
facial deformity? (description)	yes (narrow face, narrow and high palate, middle face hypoplasia and enophthalmia, hypertelorism, eye ring, low hanging columella, short philtrum, large ears)	metopic synostosis	

Face (front view)	cf. Photo		
Face (profile)	cf. Photo	plagiocephaly	
Hands	arachnodactyly, camptodactyly of the 5th finger		normal
Feet	long feet, long toes, flatfoot		normal
Other symptoms not mentioned above	marfanoid habitus: dolichostenomelia, medical history of scoliosis, heart ultrasound normal	PEG feeds, eczema, obesity	none

Supplementary Table 2. Primer sequences for qRT-PCR assay of target genes.

Gene	Forward primer	Reverse primer
Msl1	TGCCATCAAGTGTTGCAGGAG	TGGCTCAGGGAAAAATGAGGTA
Msl2	CCAGGAAGCCCTACCAAGACT	GCAGCCACGACATATACAATCT
Msl3	CCAAGTTGGGATAGATGGGCA	TGGCACGCTTTTTAAGACAGA
Cnp	AGGGCTCTGGTGGATGAATTA	CTCTGAGCGGGCATAAGGT
Mbp	GGGATCGTGCAAGGCTTCTT	GGCACCCATTTAGGAGCATTAT
Mal	AGTGGCTTCTCGGTCTTCAC	GGTAGGCTGCATCAAACTCAC
Omg	CTTCCTGCCTGTTCATCCTTC	ATCCAGGGTTCTCAGATTGGT
Kif22	GGCCGCTGTGTAAGCAAAG	TCCTTCGCTTCTGTCTCTCCA
Espl1	TCATCCTACTTCGCAATGGTTC	CTCTGCTCCCTTCCAAAACAG
Aspm	TGGCTATGAGTGAATGCTCTTCC	TCGCGTAAAAACAGTGGCAAG
Nek2	TTCCATCCTCAGCCATGAAGA	CCTGCACTTGGACTTGGCAA
Tpx2	GATGCCCCCACCGACTTTATC	CTTGTTCTCCAAGTTGGCCTT
Cdca8	CAAATTGAGTCCGACAGACAGA	GCCGAAGGATCTCGATGTTGT
Ccnb1	AAGGTGCCTGTGTGTGAACC	GTCAGCCCCATCATCTGCG
Ccnb2	GCCAAGAGCCATGTGACTATC	CAGAGCTGGTACTTTGGTGTTC
Kif18b	CAAACAGTGTCCAACCCAGAA	CAGCAGGGAGTATTGTCGC
Bub1b	GAGGCGAGTGAAGCCATGT	TCCAGAGTAAAAGCGGATTTCAG
Ccl4	CTTCTGTGCTCCAGGGTTCT	AGCAAAGACTGCTGGTCTCA
Ccl5	GCTGCTTTGCCTACCTCTCC	TCGAGTGACAAACACGACTGC
Ccl20	GCCTCTCGTACATACAGACGC	CCAGTTCTGCTTTGGATCAGC
Ccl28	GTGTGTGGCTTTTCAAACCTCA	TGCATGAACTCACTCTTTCCAG
Cxcl1	CTGGGATTCACCTCAAGAACATC	CAGGGTCAAGGCAAGCCTC
Cxcl2	CCAACCACCAGGCTACAGG	GCGTCACACTCAAGCTCTG
Cxcl10	CCAAGTGCTGCCGTCATTTTC	GGCTCGCAGGGATGATTTCAA
β-actin	GGCTGTATTCCCCTCCATCG	CCAGTTGGTAACAATGCCATGT

Supplementary Table 3. List of proteins identified by mass spectrometry in the pull down products of endogenous MSL2.

MSL2 pull down was performed from purifed OPCs and its binding partners were analyzed by mass spectrometry. Proteins identified after filtering out the IgG background are shown. Partners verified in Western blot assay were in red.

Accession	Gene Symbol	Sum PEP Score	Coverage [%]	# PSMs	# Unique Peptides	# AAs
P68373	Tuba1c	197.275	62	80	1	449
Q922F4	Tubb6	95.352	29	38	1	447
Q07231	Zscan21	73.809	32	24	16	555
Q8BFZ3	Actbl2	73.109	25	49	1	376
P17141	Zfp37	63.476	28	19	14	594
Q3UIY0	Kat8	61.547	41	14	9	334
Q62311	Taf6	57.951	23	19	13	678
Q8VE19	Mios	53.633	20	17	14	875
Q6IQX8	Znf219	51.613	23	12	11	726
P63087	Ppp1cc	51.601	34	16	1	323
P56671	Maz	43.237	16	17	6	477
Q61062	Dvl3	42.129	15	14	11	716
Q99J95	Cdk9	39.495	30	13	9	372
Q8BIF9	Znf787	38.616	32	11	8	381
P58771	Tpm1	37.941	31	12	4	284
P54818	Galc	37.287	15	12	8	684
Q80T69	Rsbn1	34.492	11	8	7	795
O35738	Klf12	34.452	20	10	4	402
Q8K1R3	Pnpt1	33.1	16	13	12	783
Q8BJ90	Znf771	32.66	30	9	8	317
P11157	Rrm2	31.788	24	9	7	390
Q60738	Slc30a1	30.901	21	8	8	503
Q3TLP5	Echdc2	28.747	30	12	7	296

Q61414	Krt15	28.422	10	18	1	452
P61963	Dcaf7	27.567	23	9	7	342
Q9WTJ4	Fiz1	27.383	15	7	5	500
P21107	Tpm3	27.252	16	9	2	285
Q7TN98	Cpeb4	25.441	11	7	6	729
Q7TQK0	Ccnt2	24.589	11	10	5	723
Q8VI33	Taf9	23.608	28	10	2	264
Q9CQV8	Ywhab	23.468	19	11	1	246
P83093	Stim2	22.917	10	6	6	746
Q9ER69	Wtap	22.834	21	6	6	396
O35492	Clk3	22.393	15	7	7	638
P08730	Krt13	22.31	11	9	1	437
P62715	Ppp2cb	21.013	20	5	5	309
Q80X73	Pelo	20.782	13	4	4	385
O35343	Kpna4	20.399	18	6	3	521
P83940	Eloc	20.275	58	5	5	112
Q8BFU2	H2ac25	19.323	35	11	2	130
Q8R2U0	Seh1l	19.228	18	6	6	360
Q3U564	Dcp1b	18.608	14	7	7	578
O35730	Ring1	18.45	10	4	2	406
A2A5Z6	Smurf2	18.33	10	10	8	748
Q5NCR9	Nsrp1	18.146	9	6	5	542
Q9JJZ6	Klf13	18.127	16	6	3	289
Q9JIY2	Cbll1	17.924	11	4	4	491
Q5DU09	Znf652	17.472	11	5	5	608
Q3UBZ5	Mif4gd	17.124	23	4	4	222
Q5NBY9	Patz1	17.056	8	10	4	687
Q61036	Pak3	16.895	9	4	4	559
P22682	Cbl	16.622	8	5	5	913

Q9CWX4	Rpusd4	16.619	19	9	7	377
Q61701	Elavl4	16.546	14	9	1	385
Q80VM4	Znf579	16.002	9	7	5	562
Q9Z315	Sart1	15.575	6	3	3	806
Q6PIE5	Atp1a2	15.265	5	4	2	1020
Q9WVB2	Tle2	15.198	7	7	1	767
O70230	Znf143	14.941	8	5	5	638
P70213	Fv1	14.352	12	3	3	459
Q8R0T2	Znf768	14.285	12	7	6	568
Q91VW9	Zkscan3	14.243	10	6	5	553
Q9R1C0	Taf7	14.236	16	6	5	341
Q9QXX4	Slc25a13	14.075	8	7	1	676
P18872	Gnao1	13.955	11	4	2	354
Q8BX09	Rbbp5	13.623	9	5	5	538
Q5RJ54	Zscan26	13.421	7	7	2	466
P08752	Gnai2	13.283	17	5	4	355
O35344	Kpna3	13.061	12	4	2	521
P14152	Mdh1	12.97	13	3	3	334
P01027	C3	12.684	3	5	5	1663
P62821	Rab1A	12.679	20	7	2	205
Q9QWV9	Ccnt1	12.52	6	3	2	724
O08789	Mnt	12.2	8	5	4	591
Q9CWW7	Cxxc1	11.95	6	3	3	660
Q8BU11	Tox4	11.948	9	4	4	619
Q8R149	Bud13	11.885	3	3	2	637
Q91X20	Ash2l	11.051	8	4	4	623
Q60722	Tcf4	10.901	5	3	3	670
Q8BUR3	Foxj3	10.845	6	3	3	623
Q99LD8	Ddah2	10.527	12	3	2	285

Q9DBC3	Cmtr1	10.347	6	6	5	837
Q80US4	Actr5	10.128	7	4	3	605
P62046	Lrch1	10.025	4	2	2	709
Q3UN04	Usp30	9.828	6	5	3	517
Q9ERG0	Lima1	9.768	4	3	3	753
Q3UFS4	Gpatch11	9.732	10	5	4	262
Q8CB77	Eloa	8.871	3	2	2	773
Q6PIP5	Nudcd1	8.656	4	2	2	582
Q8K4Q6	Neil1	8.519	7	2	2	389
Q31125	Slc39a7	8.473	3	2	1	476
Q8R409	Hexim1	8.423	10	2	2	356
Q3US17	Znf48	8.176	9	4	3	591
Q8C092	Taf5	8.068	3	2	2	801
Q8BG07	Pld4	8.05	5	2	2	503
P30416	Fkbp4	8.033	6	2	2	458
Q6P5F6	Slc39a10	7.893	2	4	2	833
O70194	Eif3d	7.868	7	3	3	548
Q62511	Zfp91	7.864	4	3	2	572
Q04888	Sox10	7.846	19	9	3	466
P61089	Ube2n	7.597	14	3	2	152
P14211	Calr	7.581	7	3	3	416
Q05CL8	Larp7	7.533	4	2	2	570
Q8R2S9	Actr8	7.468	7	5	4	624
Q8BFY9	Tnpo1	7.364	3	2	2	898
Q8R123	Flad1	7.33	6	2	2	492
Q8BQN6	Znf146	7.073	8	2	2	292
P42225	Stat1	7.007	3	2	2	749
Q9JKK7	Tmod2	6.978	8	3	2	351
Q9JHJ0	Tmod3	6.942	8	3	2	352

Q99JT2	Stk26	6.869	5	3	2	416
Q62513	Znf260	6.864	6	3	2	407
Q8BFR1	Zc3hav1l	6.859	9	3	3	296
O88544	Cops4	6.214	5	2	2	406
Q9CS42	Prps2	6.189	6	2	2	318
Q8VE65	Taf12	6.14	18	3	3	161
Q02614	Sap30bp	6.135	6	2	2	308
Q9Z2D6	Mecp2	5.919	8	3	3	484
Q8BW56	Gtdc1	5.897	6	2	2	445
P56873	Znrd2	5.831	9	2	2	199
Q922M3	Kctd10	5.719	6	2	2	315
Q9DBS1	Tmem43	5.635	6	2	2	400
Q9Z1Q5	Clic1	5.595	9	2	2	241
Q9DB38	Znf580	5.554	13	2	2	172
Q6PAC3	Dcaf13	5.48	4	3	2	445
Q99NB9	Sf3b1	5.475	2	2	2	1304
Q9DB94	Wdr53	5.286	6	2	2	358
Q9CQI7	Snrpb2	5.254	15	3	1	225
Q9CX56	Psmd8	5.204	7	2	2	353
Q810A7	Ddx42	5.203	2	2	2	929
A2AIL4	Ndufaf6	5.073	8	2	2	333
P63001	Rac1	4.97	11	2	2	192
Q08376	Zbtb14	4.925	4	2	2	449
Q99L47	St13	4.922	8	2	2	371
Q99PM3	Gtf2a1	4.873	5	2	2	378
Q60980	Klf3	4.871	5	3	1	344
P46660	Ina	4.713	4	2	1	501
Q80U70	Suz12	4.461	4	2	2	741
Q99J45	Nrbp1	4.423	6	2	2	535

P62814	Atp6v1b2	4.402	5	3	2	511
P22892	Ap1g1	4.273	2	2	2	822
P13020	Gsn	4.076	2	2	2	780
Q99PT3	Ino80b	3.986	5	2	2	375
Q6IRU5	Cltb	3.803	4	2	2	229
Q5U458	Dnajc11	3.605	4	2	2	559
Q8BK63	Csnk1a1	3.604	8	2	2	337
Q8BY02	Nkrf	3.565	3	2	2	690
O08908	Pik3r2	3.445	3	2	2	722
P50396	Gdi1	3.146	6	2	2	447
P25916	Bmi1	3.066	5	3	2	324
Q69ZF8	Msl2	3.065	8	7	3	577
Q571J5	Znf354c	3.056	4	2	2	560
Q99JI6	Rap1b	3.007	12	2	2	184
Q5XJY5	Arcn1	3.005	3	2	2	511
Q9JKN5	Olig1	2.947	8	4	1	260
O88543	Cops3	2.519	7	2	2	423
Q6PDM1	Msl1	2.35	5	2	2	616
Q61382	Traf4	2.108	3	2	2	470
Q9CRB0	Snx24	1.46	4	2	1	169
P46096	Syt1	1.281	7	1	1	421

Supplementary Figures

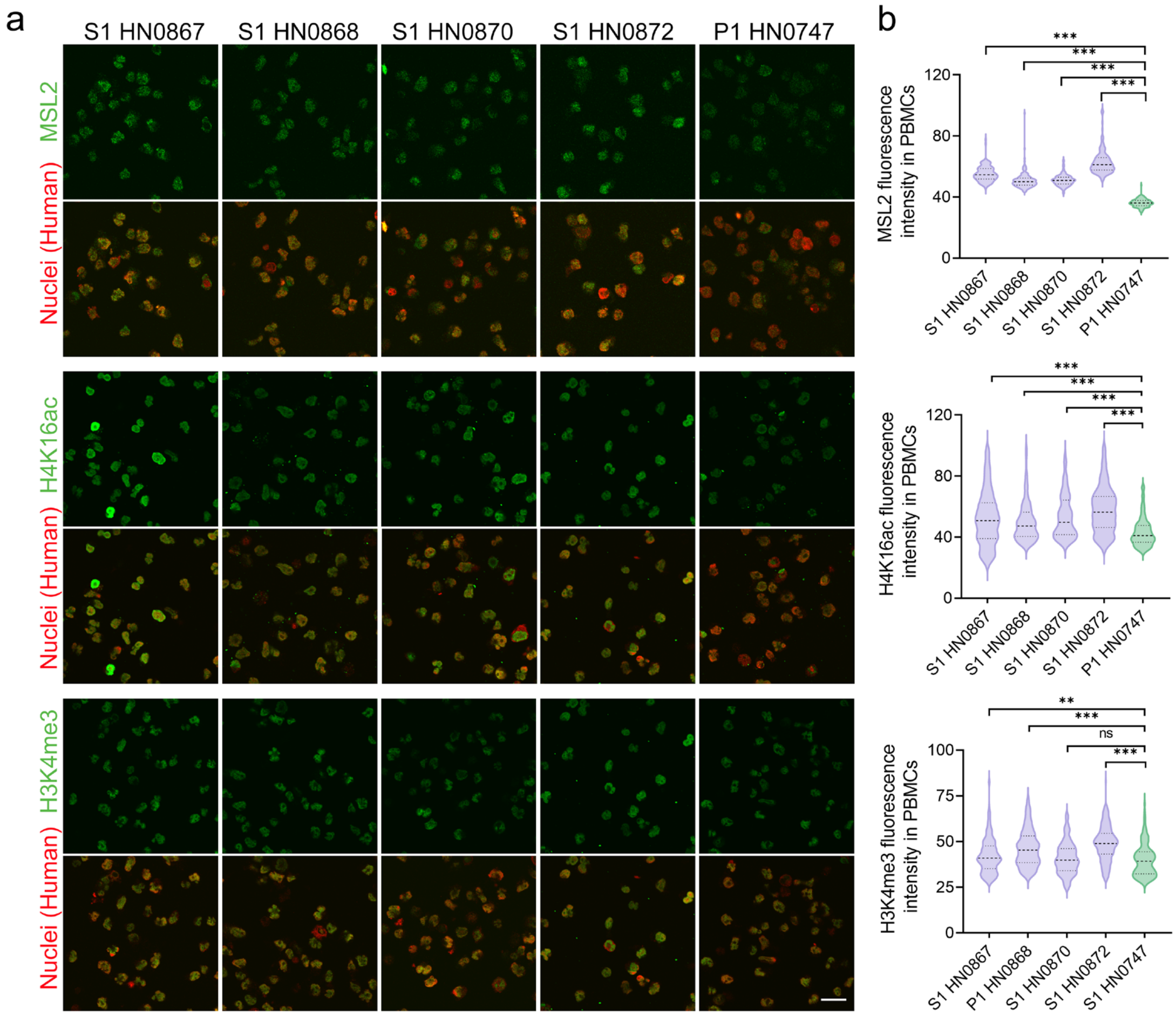

Supplementary Fig.1 Immunostaining of MSL2, H4K16ac and H3K4me3 in immortalized lymphocytes from patient and controls

a Representative image as indicated. Scale bar, 20 μm. **b** Quantification the fluorescence intensity. Data were Mean ± SEM (n=120~200 cells in each group). One-Way ANOVA, $**p < 0.01$, $***p < 0.001$.

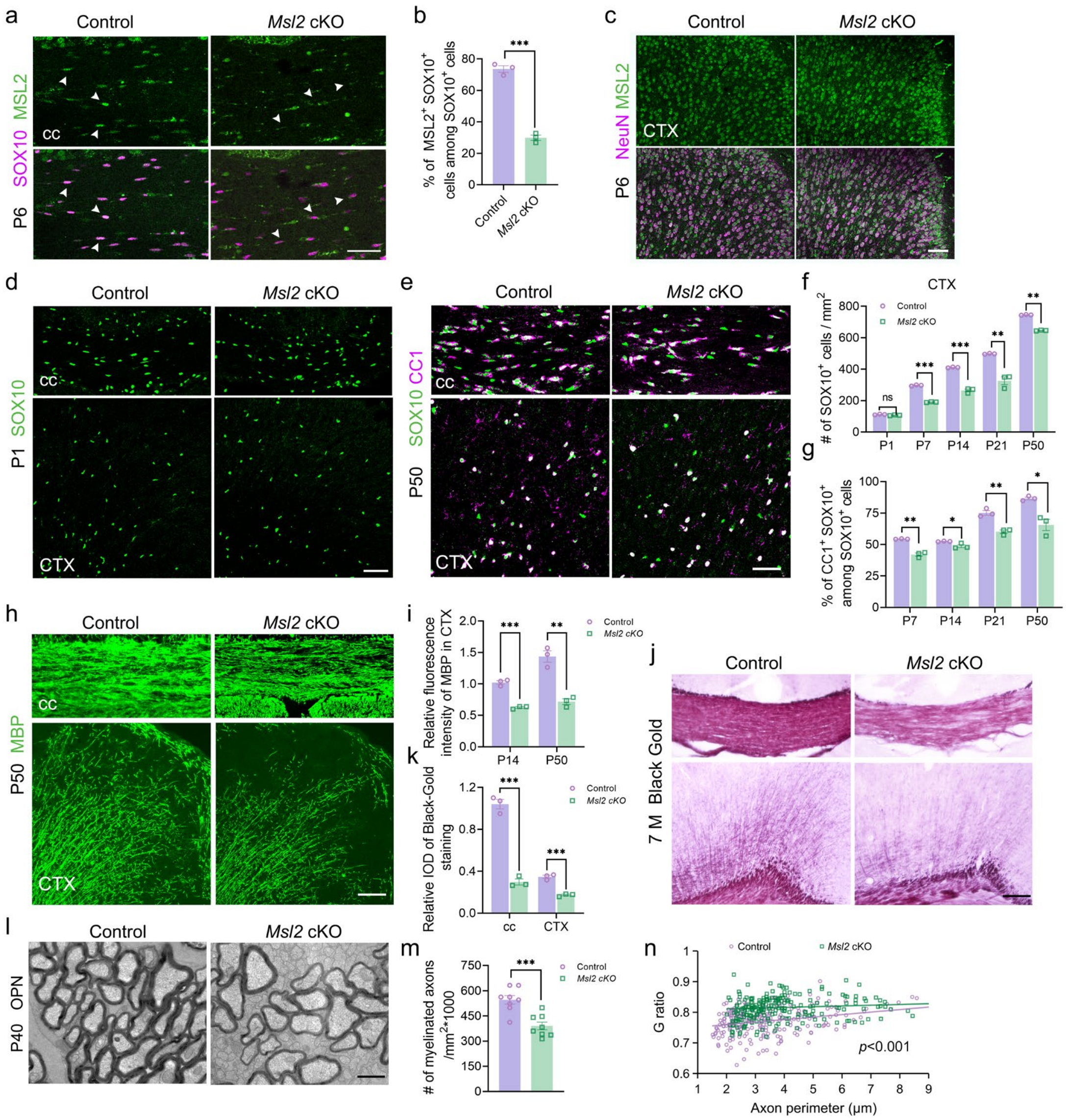

Supplementary Fig.2 MSL2 is required for OL differentiation and myelination

a Representative immunostaining of MSL2 and SOX10 in cc from P6 control and *Msl2* cKO mice. Arrowheads indicate SOX10$^+$ cells. Scale bar, 100 μm. **b** Quantification the proportion of MSL2$^+$ cells among SOX10$^+$ cells in cc from P6 control and *Msl2* cKO mice. Data were Mean ± SEM (n = 3 animals each group). Two tail unpaired t-test, ****p* < 0.001. **c** Representative immunostaining of NeuN and MSL2 in cortex from P6 control and *Msl2* cKO mice. Scale bar, 100 μm. **d** Representative immunostaining for SOX10 in cc and CTX from P1 control and *Msl2* cKO mice. Scale bar, 100 μm. **e** Representative immunostaining for CC1 and SOX10 in cc and CTX from P50 control and *Msl2* cKO mice. Scale bar, 100 μm. **f** Quantification the density of SOX10$^+$ cells in CTX from control and *Msl2* cKO mice at different ages. Data were Mean ± SEM (n = 3 animals each group). Two tail unpaired t-test, n.s. (not significant), *p* > 0.05, ***p* < 0.01, ****p* < 0.001. **g** Quantification the proportion of SOX10$^+$ cells in CTX from control and *Msl2* cKO mice at different ages. Data were Means ± SEM (n = 3 animals each group). Two tail unpaired t-test, **p*< 0.05, ***p* < 0.01. **h** Representative immunostaining for MBP in cc

and CTX from control and *Msl2* cKO mice at P50. Scale bar, 100 µm. **i** Quantification the relative intensity of MBP staining in CTX of control and *Msl2* cKO mice at P14 and P50. Data were Mean ± SEM (n = 3 animals each group). Two tail unpaired t-test, ***p <0.001. **j** Representative images of black gold staining for myelin in cc and CTX of control and *Msl2* cKO mice at 7 months. Scale bar, 100 µm. **k** Quantification the integrated optical density (IOD) of black gold staining of control and *Msl2* cKO mice at 7 months. Data were Mean ± SEM (n =3 animals each group). Two tail unpaired t-test, ***p <0.001. **l** Representative electron micrographs of P40 optic nerve from control and *Msl2* cKO mice. Scale bar, 1 µm. **m** Quantification the number of myelinated axons in defined areas from the optic nerve of P40 control and *Msl2* cKO mice. Data were Means ± SEM (n = 8 slides from three animals per group). Two-tailed unpaired t-test, ***p <0.001. **n** G ratios vs. axonal perimeters in optic nerve from P40 control and *Msl2* cKO mice. Mann Whitney test, *p <0.05 (Control, n=230; *Msl2* cKO, n=231 myelinating axon counted from three animals each genotype).

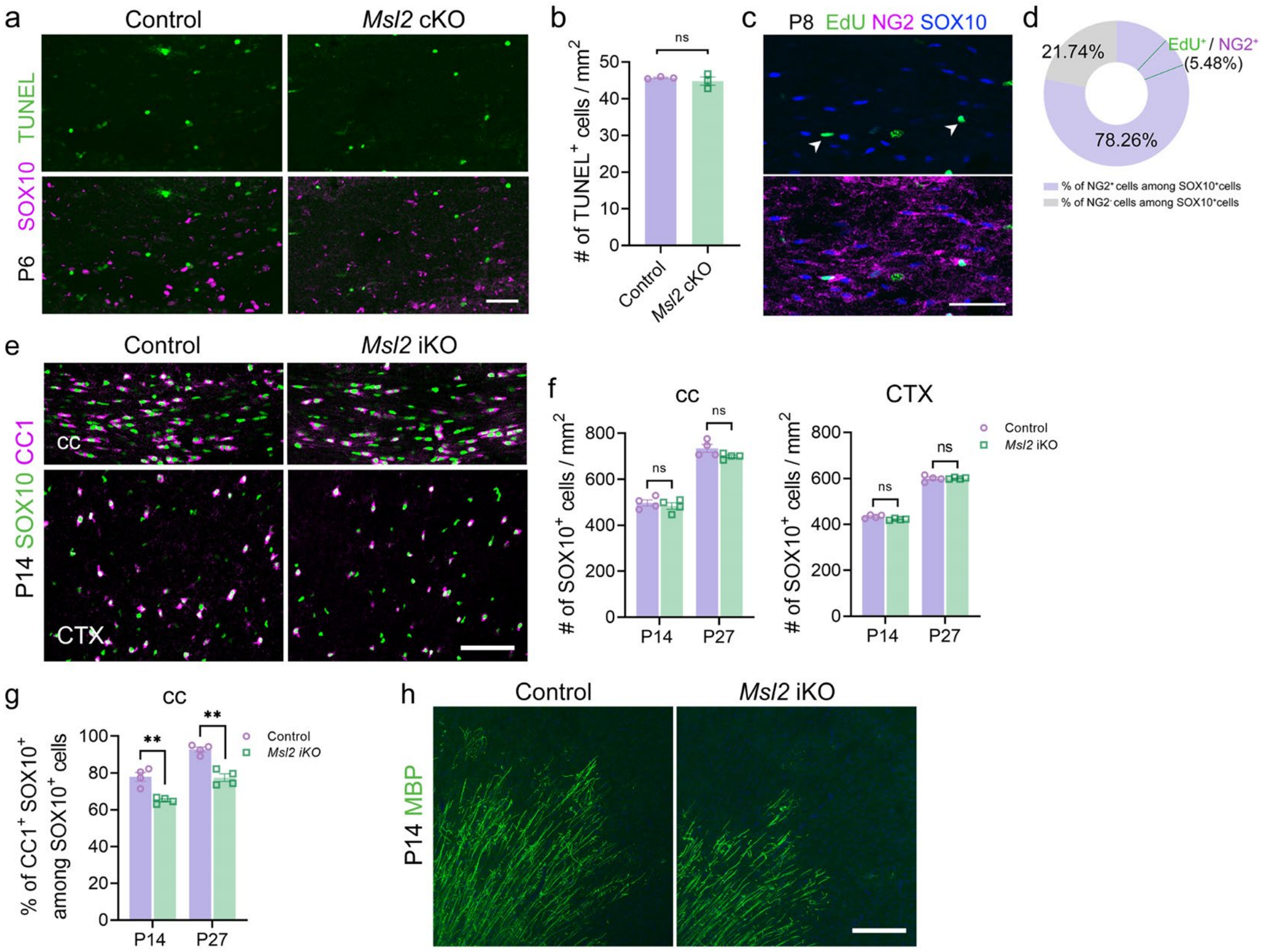

Supplementary Fig.3 *Msl2* iKO mice recapitulate the hypomyelination phenotype as in *Msl2* cKO mice

a Double staining of SOX10 and TUNEL in cc from P6 control and *Msl2* cKO mice. Scale bar, 100 µm. **b** Quantification the density of TUNEL$^+$ cells. Data were Means ± SEM (n = 3 animals each group). Two tail unpaired t - test, n.s. (not significant), $p > 0.05$. **c** Representative immunostaining of EdU, NG2 and SOX10 in cc from P8 wild type mice. Scale bar, 50 µm. **d** Quantification the percentage of NG2$^+$ cells among SOX10$^+$ cells and the percentage of EdU$^+$ cells among NG2$^+$ populations. **e** Representative immunostaining of CC1 and SOX10 in cc and CTX from P14 control and iKO mice. Scale bar, 100 µm. **f** Quantification the density of SOX10$^+$ cells in cc and CTX from P14 and P27 control and iKO mice. Data were Means ± SEM (n = 4 animals each group). Two tail unpaired t - test, n.s. (not significant), $p > 0.05$. **g** Quantification the percentage of CC1$^+$ cells among SOX10$^+$ cells in CTX from P14 and P27 control and iKO mice. Data were Means ± SEM (n = 3 animals each group). Two tail unpaired t - test. *** $p < 0.001$. **h** Representative immunostaining of MBP in CTX from P14 control and iKO mice. Scale bar, 100 µm.

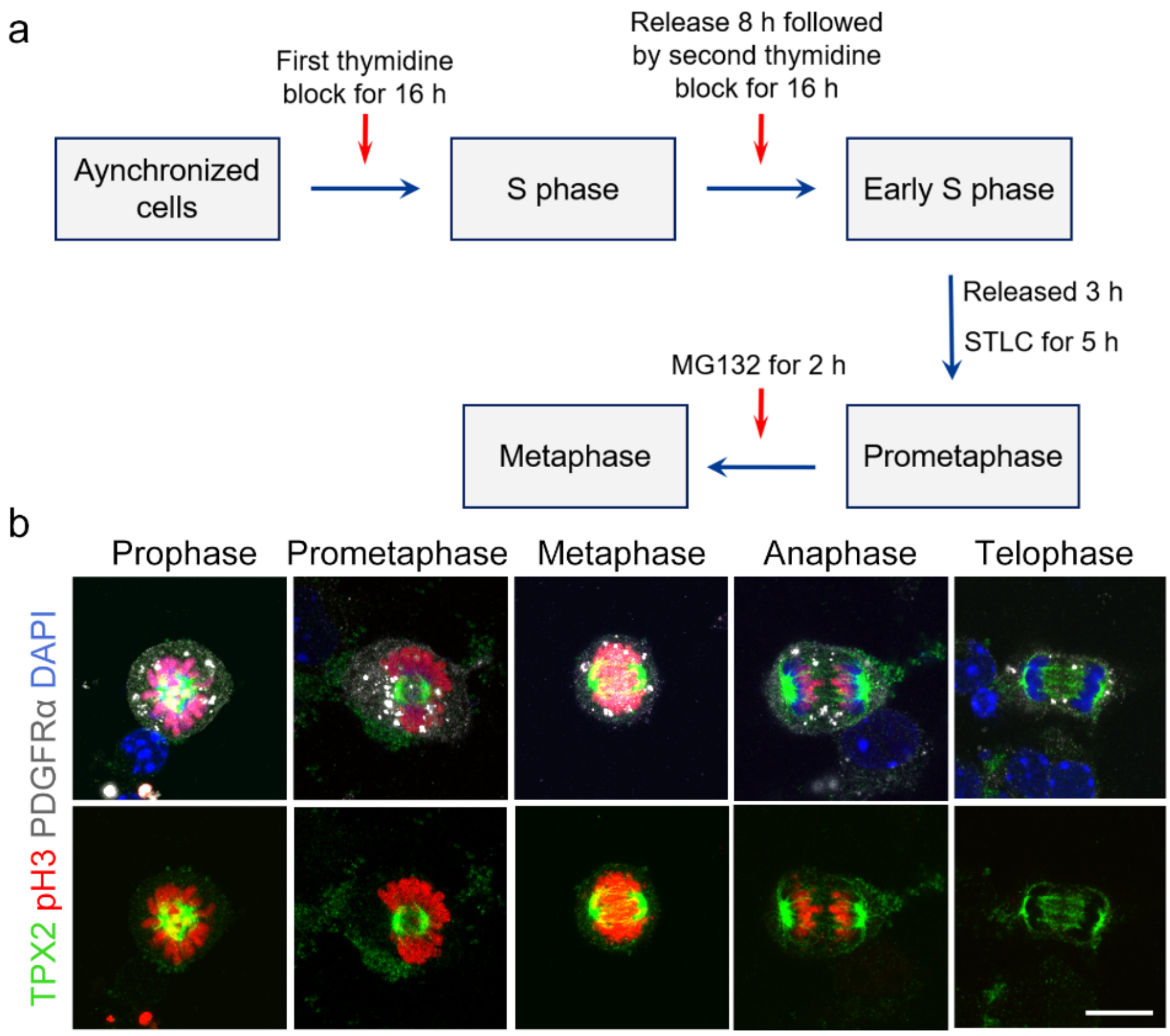

Supplementary Fig.4 Assessment of cell synchronization for OPC cultures

a Flow chart to show the steps of cell synchronization to metaphase in purified OPC cultures. **b** Staining of TPX2 and pH3 in OPC cultures to show spindle shape in various mitotic stage. Scale bar, 10 μm.

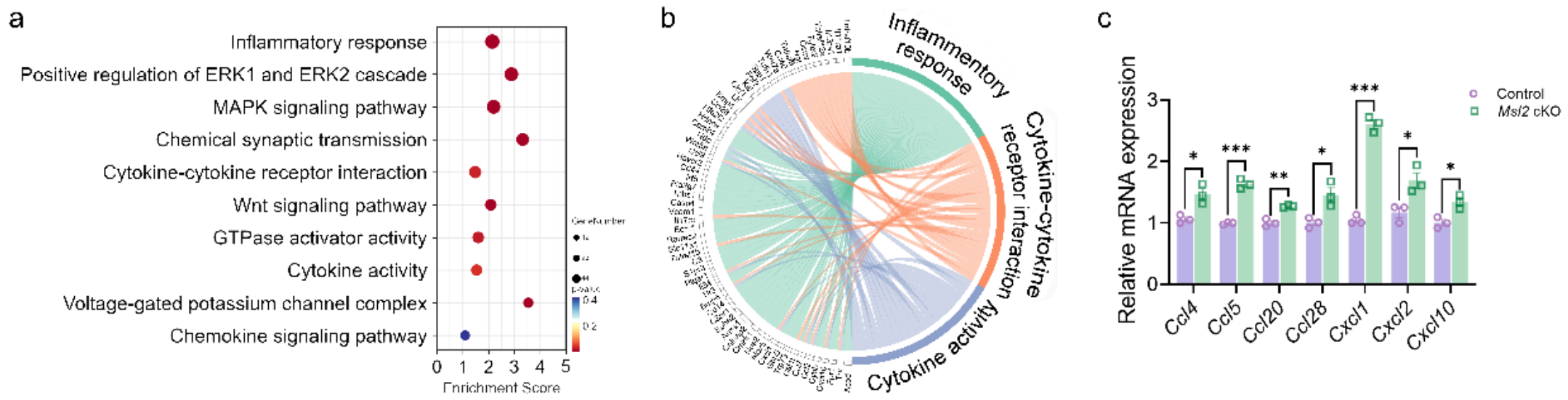

Supplementary Fig.5 *Msl2* ablation induces upregulation of several cytokine transcripts in OPCs

a Bubble plots of GO analysis for upregulated in *Msl2* cKO mice. **b** Circos diagram of representative GO terms, inflammatory response, cytokine-cytokine receptor interaction and cytokine activity, for upregulated genes in *Msl2* cKO mice. **c** qRT-PCR validation of representative upregulated inflammatory genes in *Msl2* cKO OPCs. Data were Mean±SEM of transcript levels relative to control after normalization (n = 3 independent cultures each performed in triplicate). Two tail unpaired t-test, *p <0.05; **p <0.01; ***p <0.001.

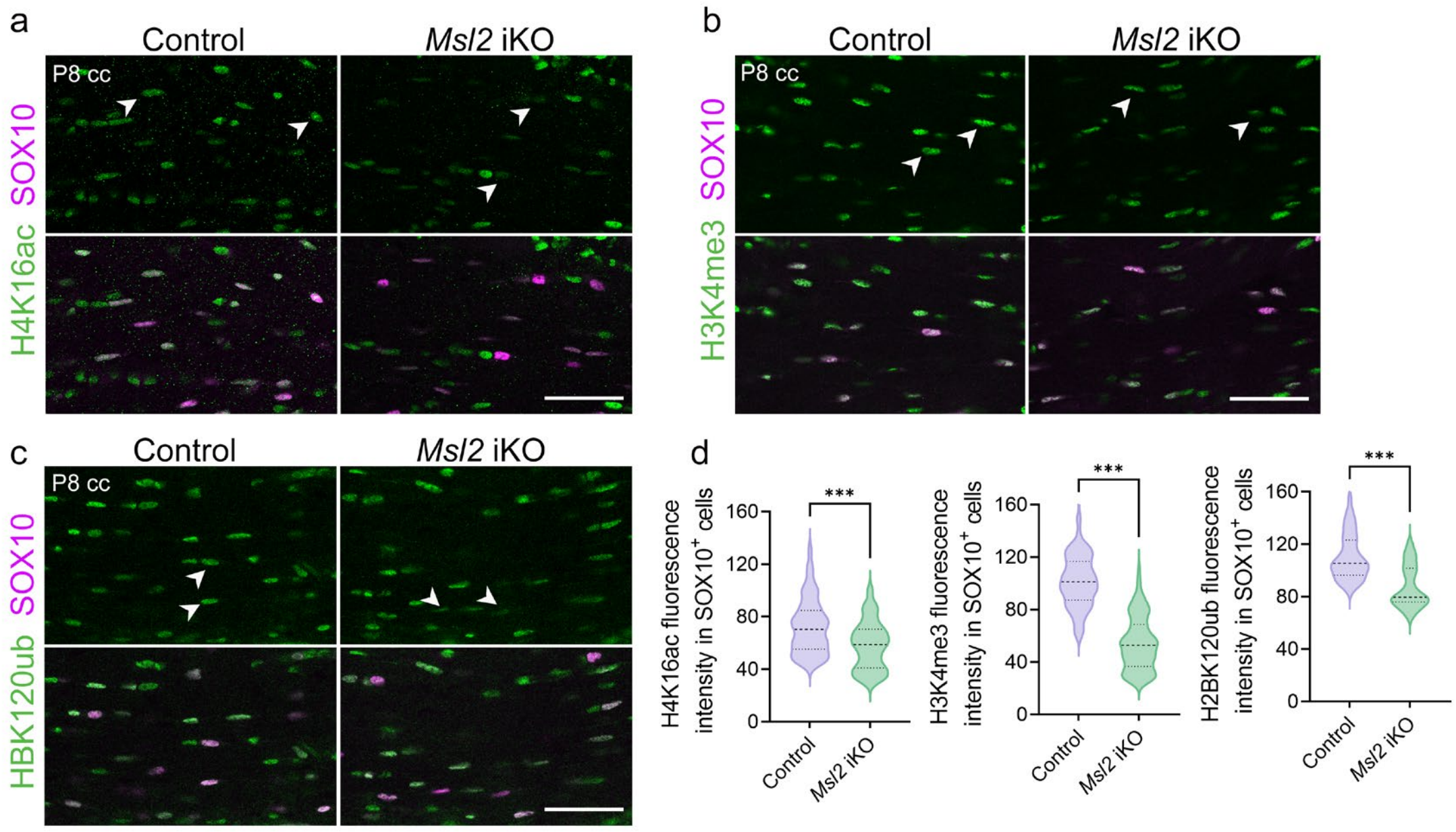

Supplementary Fig.6 Immunostaining of H4K16ac, H3K4me3 and H2BK120ub in P8 *Msl2* iKO mice.

a-c Representative images as indicated. Scale bar 50 μm. **d** Quantification of immunofluorescence intensity in $SOX10^+$ cells. Data were Mean ± SEM (n=54~157 cells in each group). Two tail unpaired t-test, ****p* < 0.001.

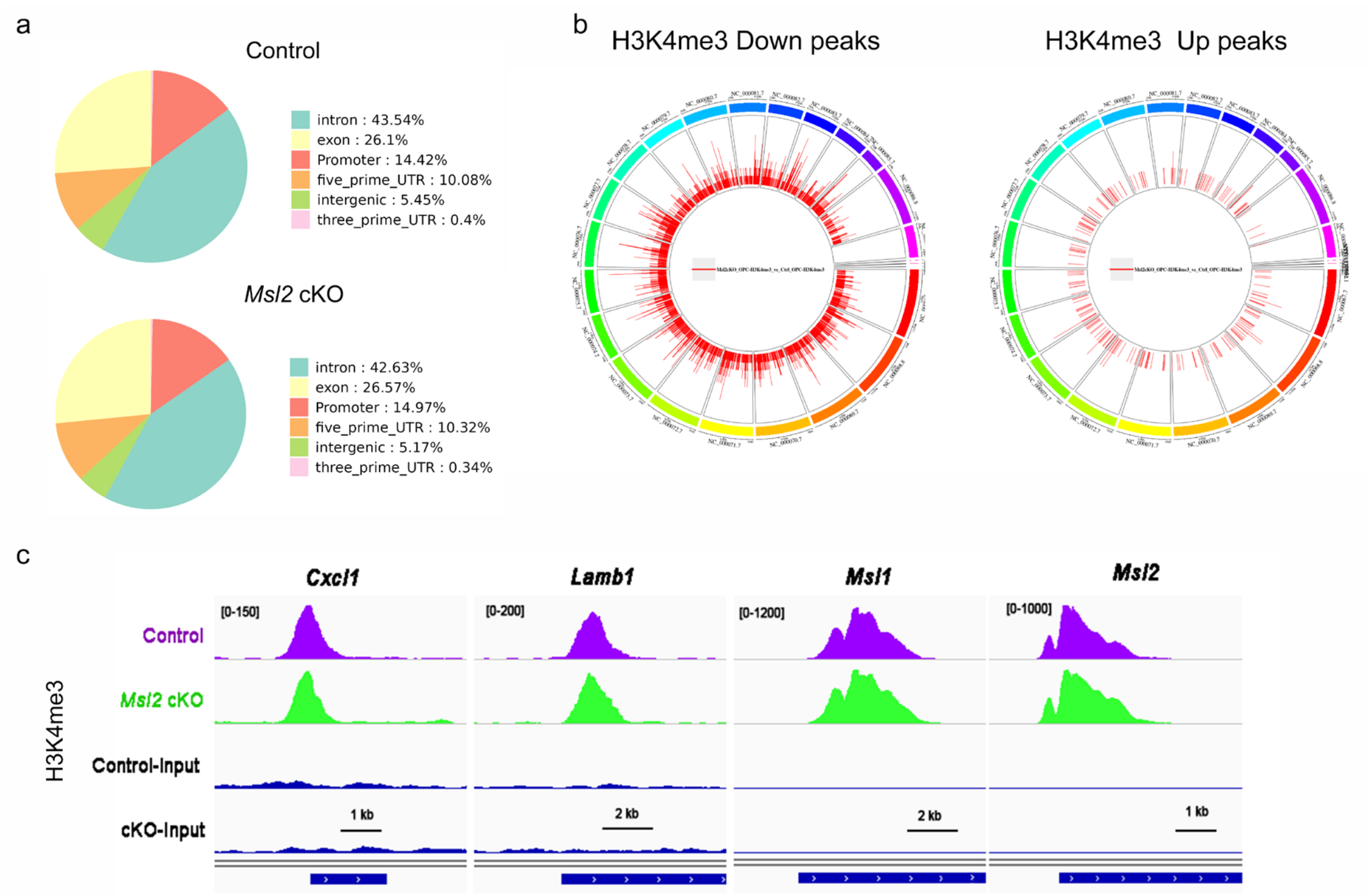

Supplementary Fig.7 H3K4me3 profiles in *Msl2* ablated OPCs

a Pie charts depicting the annotations of genomic locus distribution of H3K4me3 peaks from control and *Msl2* cKO group. **b** Genome map of the distribution of H3K4me3 peaks. **c** Snapshots of H3K4me3 profiles in constantly expressed genes from OPCs after *Msl2* ablation. Tracks were shown in IGV 2.4.8.

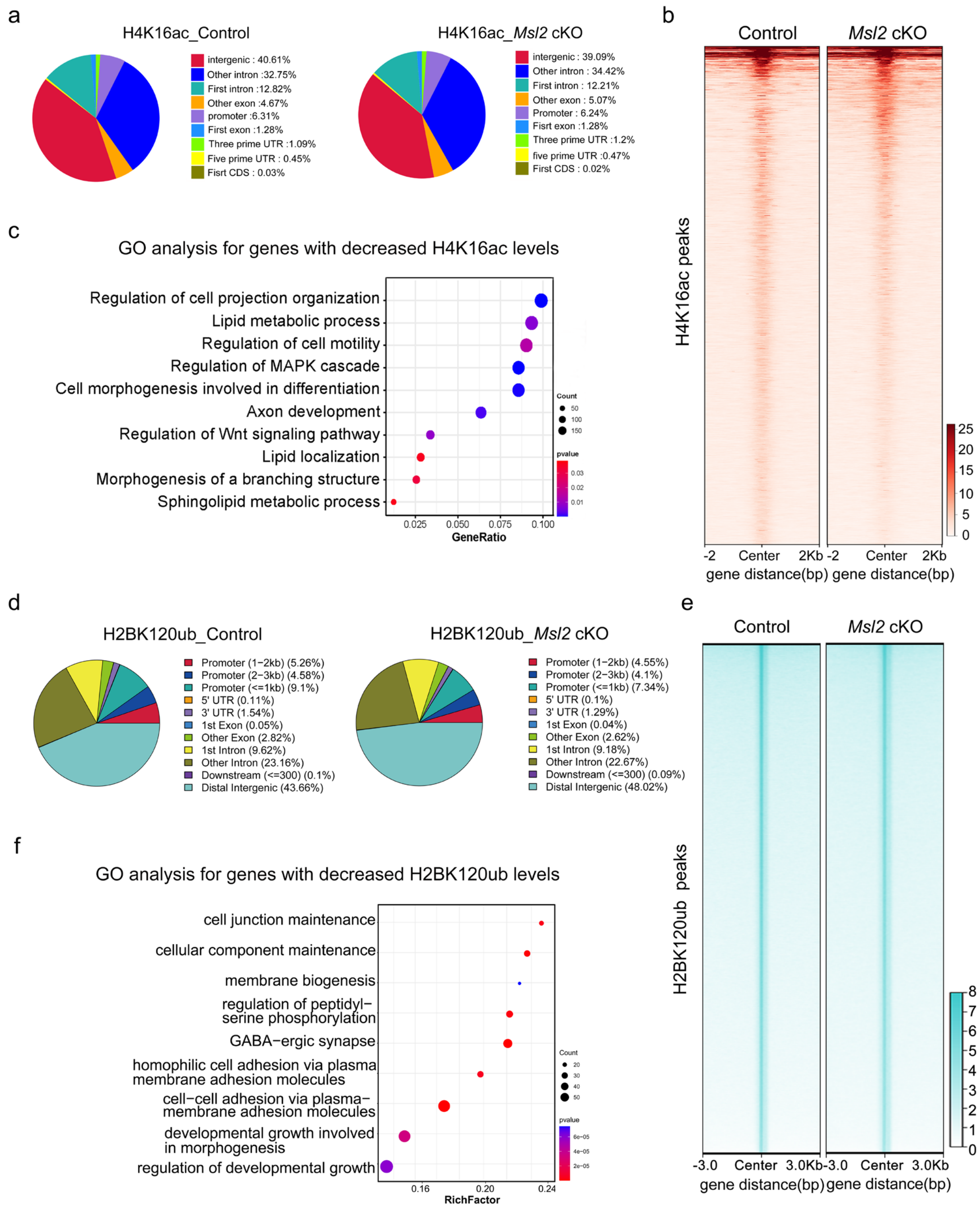

Supplementary Fig.8 H4K16ac and H2BK120ub profiles in *Msl2* ablated OPCs

a Pie charts depicting the annotations of genomic locus distribution of H4K16ac peaks from control and *Msl2* cKO group. **b** Heatmaps depicting H4K16ac peaks signals in control and *Msl2* cKO group. **c** GO analysis for genes with decreased H4K16ac levels in *Msl2* cKO OPCs. **d** Pie charts depicting the annotations of genomic locus distribution of H2BK120ub peaks from control and *Msl2* cKO group . **e** Heatmaps depicting H2BK120ub peaks signals in control and *Msl2* cKO group . **f** GO analysis for genes with decreased H2BK120ub levels in *Msl2* cKO OPCs.

Supplementary Materials and Methods

Cell Synchronization

To be synchronized in metaphase for immunofluorescence, purified OPCs were first treated with double thymidine (Selleck, S4803), first thymidine block for 16 h, released 8 h followed by second thymidine block for 16 h. After release for 8 h, cells were incubated with STLC (S-Trityl-L-cysteine) (Selleck, S5522) for 8 h to induce monopolar spindle formation. These cells were then released into fresh medium containing MG132 (carbobenzoxy-Leu-Leu-leucinal) (Selleck, 2619) for 2 h to allow full establishment of bipolar spindles [1].

Quantitative RT-PCR

For qRT-PCR, RNA was transcribed to cDNA with the PrimeScript™ RT reagent Kit (Perfect Real Time, Takara RR037A) and reactions were conducted with LightCycler® RNA Master SYBR Green I (Roche 03064760001) in CFX96 Touch Real-Time PCR Detection System (Bio-Rad). Relative gene expression was normalized to internal control β-actin. Primer sequences for target genes are listed in Supplementary Table 2.

Western Blot Analysis

Western blot assay was carried out as previously described[2].The following primary antibodies were used: SOX10 (Santa Cruz 17342), MSL2 (Cell Signaling Technology, #4406), MSL2 (Invitrogen, # PA5-88985), H4K16ac (Abcam ab109463), H3K4me3 (Cell Signaling Technology, #9751), H2BK120ub (PTM BIO, #PTM-1122), H2AK119ub (PTM BIO, #PTM-7449), KAT8 (Proteintech,13842-1-AP), H3 (Proteintech,17168-1-AP), H4 (PTM BIO, #PTM-1009).

EdU (5-ethynyl-2'-deoxyuridine) labeling

For EdU labelling *in vivo*, mice were intraperitoneally injected with 25 mg/kg EdU at P7, and executed at 8 and 48 hrs later, respectively. EdU staining was performed following the manual (Beyotime, C0071L) and co-staining protocols were performed

as described above.

Mouse Behavioral Assays

All behavioral experiments were performed during the light cycle (9:00 a.m. to 20:00 p.m.) in a sound proof room, and adolescent mice and adult mice were used for behavioral tests in our study. Two groups of mice were tested respectively and less stressful behaviors were tested first, with more stressful procedures at the end (Fig. 5a).

Three-chamber test

Three-chamber test was used to test the social approach and social novelty preference in mice[3]. The time spent in each side chamber was recorded and analyzed using SMART v.3.0 software (Panlab, Harvard Apparatus).

Resident–intruder test

Resident-intruder tests were carried out according to previous report[4]. Resident mice were individually housed for one week before testing and intruder mice of the same age and sex were placed in the resident cage. The social behavior between the mice, including approach, olfactory touch, hair, chase, attack, escape, were recorded and the time of social interaction was measured manually.

Grooming

The self-grooming behavior test was adapted from previously published work [5]. Mice were placed individually in a test chamber (25 cm × 25 cm × 30 cm) with 35 lux illumination. The mice acclimated to the test chamber for 3-5 min, and then the spontaneous activities were recorded for 30 min.

Open Field Test (OFT)

Mice were placed close to the corner of open field (50 cm × 50 cm × 40 cm) and left to explore freely for 5 min. The anxiety level of each mouse was evaluated by their activities in the arena's central region (25%) as previously reported[6].

Elevated plus maze (EPM)

Mice were placed on the central platform of EPM and allowed to move freely for 5 min. The SMART3.0 tracking system was used to analyze the data, and the percentage of time and distance traveled in the open arms is calculated to assess anxiety level as reported in previous study[7].

Tail Suspension Test (TST)

The tail-suspension test is commonly used to assess depression-related behaviors [8]. The mice were suspended downward by fixing 1/3 tail with tapes. The struggling and immobility behaviors were recorded for 10 min, and the immobility time within the next 6 min was counted for analysis.

Sucrose Preference Test (SPT)

As reported in previous studies[9], the sucrose preference was calculated using the formula: [Vol of sucrose / Vol of total liquid] × 100%.

Novel object recognition test (NOR)

In the training phase of novel object recognition test, each mouse was individually allowed to explore two identical objects for 10 min, which was placed 5 cm from a corner. Two hours later, during the test trial, one of the familiar objects was replaced with a novel object, and the exploration was continued for 10 min. The exploration was defined as the mouse touching the object with the tip of its nose or the front paws. The discrimination ratio was calculated to measure the recognition memory of each mouse and was computed as follows:

$$Preference\ index\ (PI) = \frac{Time_{(Novel)} - Time_{(Familiar)}}{Time_{(Novel)} + Time_{(Familiar)}}$$

RNA seq and Data Analysis

Total RNA extraction from control and *Msl2* cKO OPC cultures was performed using TRIzol. The RNA libraries were sequenced on the illumina NovaseqTM 6000

platform by OE Biotech, Inc. (Shanghai, China). The DEGs were identified using Cuffdiff with p value ≤ 0.05 and further screened by RPKM $|\log_2 \text{fold}| \geq 0.58$. TopHat output data were then analyzed by DEGseq (1.22.2) package to compare the changes of gene expression between *Msl2* cKO and control, based on the calculated RPKM values for known transcripts in mouse genome reference. Bioinformatic analysis was performed using the OECloud tools at https://cloud. oebiotech.com/task/ based on the R Package (https://www.r-project.org/).

Immunoprecipitation and Mass-Spectrometry

Protein extractions from purified OPC cultures were performed for immunoprecipitation assay with the Catch and Release v2.0 Reversible Immunoprecipitation System (Millipore, 17-500). Rabbit anti-MSL2 antibody (Cell Signaling Technology, #44006,1:50) was used and normal rabbit IgG (Proteintech,30000-0-AP) were used as negative control for IP. After destaining and shrinking, the SDS-PAGE gel was treated with 10 mM DTT for protein reduction, followed by 50 mM iodoacetamide (IAA) treatment for alkylation. Protein digestions were performed with 0.02 μg/μl trypsin at 37°C overnight and the samples were then desalted for LC–MS/MS analysis (Ultimate 3000nano/Exactive plus, Thermo Fisher Scientific). Liquid chromatography-mass spectrometry was performed by Shanghai OE Biotech Co., Ltd. Proteins were identified using ProteomeDiscover 2.5 software.

Supplementary References

1 Wang, Z. *Cell-Cycle Synchronization: Methods and Protocols*. (Science+Business Media, 2022).

2 Zhang, M. *et al.* ITPR2 Mediated Calcium Homeostasis in Oligodendrocytes is Essential for Myelination and Involved in Depressive-Like Behavior in Adolescent Mice. *Adv Sci (Weinh).* **11**, e2306498 (2024).

3 Wang, Q. *et al.* Impaired calcium signaling in astrocytes modulates autism spectrum disorder-like behaviors in mice. *Nat Commun.* **12**, 3321 (2021).

4 Nakamoto, C. *et al.* GluD1 knockout mice with a pure C57BL/6N background show impaired fear memory, social interaction, and enhanced depressive-like behavior. *PLoS One.* **15**, e0229288 (2020).

5 Ge, J. *et al.* Ventral zona incerta parvalbumin neurons modulate sensory-induced and stress-induced self-grooming via input-dependent mechanisms in mice. *iScience.* **27**, 110165 (2024).

6 Kraeuter, A. K., Guest, P. C. & Sarnyai, Z. The Open Field Test for Measuring Locomotor Activity and Anxiety-Like Behavior. *Methods Mol Biol.* **1916**, 99-103 (2019).

7 Kraeuter, A. K., Guest, P. C. & Sarnyai, Z. The Elevated Plus Maze Test for Measuring Anxiety-Like Behavior in Rodents. *Methods Mol Biol.* **1916**, 69-74 (2019).

8 Can, A. *et al.* The tail suspension test. *J Vis Exp.* 10.3791/376910.3791/3769, e3769 (2012).

9 Li, Y. *et al.* The Eph receptor A4 plays a role in demyelination and depression-related behavior. *J Clin Invest.* **132** (2022).
