# Supplementary material for: MSL2 orchestrates oligodendrocyte precursor cell cycle via H3K4me3 to prevent white matter defects in neurodevelopmental disorders
Source: Exp Mol Med. 2026 Oct 6;58(10):3472–85. doi: 10.1038/s12276-026-01858-1 (PMC13736987; doi:10.1038/s12276-026-01858-1)
Supplement: Supplementary file 2 [file 12276_2026_1858_MOESM2_ESM.docx]

UDN-AUS: Collaborative Group List

The Undiagnosed Disease Network Australia collaborative group is

Alison Yeung. Victorian Clinical Genetics Services, Melbourne, VIC, Australia. (alison.yeung@vcgs.org.au)

Andrew Paul Fennell. Monash Health, Melbourne, VIC, Australia. (andrew.fennell@monashhealth.org)

Anna Hackett. Genetics of Learning Disability Service, Newcastle, NSW, Australia. (Anna.Hackett@health.nsw.gov.au)

Anne Baxter. Hunter Genetics, Newcastle, NSW, Australia. (Anne.Baxter@health.nsw.gov.au)

Ansley Morrish. Sydney Children's Hospital Network, Sydney, NSW, Australia. (ansley.morrish@health.nsw.gov.au)

Ashil Davawala. Victorian Clinical Genetics Services, Melbourne, VIC, Australia. (ashil.davawala@vcgs.org.au)

Azure Hermes. Australian National University, Canberra, ACT, Australia. (azure.hermes@anu.edu.au)

Ben Kamien. Genetic Health WA, Perth Children's Hospital, WA, Australia. (Benjamin.Kamien@health.wa.gov.au)

Ben Lundie. Pathology Queensland, Royal Brisbane & Women's Hospital, Brisbane, QLD, Australia. (ben.lundie@health.qld.gov.au)

Carolyn Ellaway. Sydney Children's Hospital Network, Sydney, NSW, Australia. (carolyn.ellaway@health.nsw.gov.au)

Carolyn Shalhoub. Sydney Children's Hospital Network, Sydney, NSW, Australia. (carolyn.shalhoub@health.nsw.gov.au)

Cas Simons. Centre for Population Genomics, Garvan Institute of Medical Research and UNSW Sydney, Sydney, NSW, Australia. (cas.simons@populationgenomics.org.au)

Cassandra Gray. Centre for Population Genomics, Garvan Institute of Medical Research and UNSW Sydney, Sydney, NSW, Australia. (cassie.gray@populationgenomics.org.au)

Cathryn Poulton. Undiagnosed Diseases Program, Genetic Health WA and Rare Care Centre, Perth Children's Hospital. (cathryn.poulton@health.wa.gov.au)

Chloe Cunningham. Murdoch Children's Research Institute, Melbourne, VIC, Australia. (chloe.cunningham@vcgs.org.au)

Chris Barnett. Women's and Children's Hospital, Adelaide, SA, Australia. (christopher.barnett@sa.gov.au)

Christopher Richmond. Genetic Health Queensland, Royal Brisbane & Women's Hospital, Brisbane, QLD, Australia. (chris.richmond@health.qld.gov.au)

Christopher Richards. Centre for Population Genomics, Garvan Institute of Medical Research and UNSW Sydney, Sydney, NSW, Australia. (christopher.richards@populationgenomics.org.au)

Clara Chung. Sydney Children's Hospital Network, Sydney, NSW, Australia. (clara.chung@health.nsw.gov.au)

Daniel G. MacArthur. Centre for Population Genomics, Garvan Institute of Medical Research and UNSW Sydney, Sydney, NSW, Australia. (daniel.macarthur@populationgenomics.org.au)

Daniel Pavlic. Centre for Population Genomics, Garvan Institute of Medical Research and UNSW Sydney, Sydney, NSW, Australia. (daniel.pavlic@populationgenomics.org.au)

Daniella Hock. The University of Melbourne, Melbourne, VIC, Australia. (daniella.hock@unimelb.edu.au)

Daniz Kooshavar. Murdoch Children's Research Institute, Melbourne, VIC, Australia. (daniz.kooshavar@mcri.edu.au)

David Amor. Victorian Clinical Genetics Services, Melbourne, VIC, Australia. (david.amor@mcri.edu.au)

David Mowat. Sydney Children's Hospital Network, Sydney, NSW, Australia. (david.mowat@health.nsw.gov.au)

David A. Stroud. Murdoch Children's Research Institute, Melbourne, VIC, Australia. (david.stroud@unimelb.edu.au)

Edward Formaini. Centre for Population Genomics, Garvan Institute of Medical Research and UNSW Sydney, Sydney, NSW, Australia. (edward.formaini@populationgenomics.org.au)

Elaine Zhang. Murdoch Children's Research Institute, Melbourne, VIC, Australia. (elaine.zhang@mcri.edu.au)

Ella Jane Wilkins. Murdoch Children's Research Institute, Melbourne, VIC, Australia. (ella.wilkins@vcgs.org.au)

Ella Zurita. Sydney Children's Hospital Network, Sydney, NSW, Australia. (Ella.Zurita@health.nsw.gov.au)

Ellenore M. Martin. Murdoch Children's Research Institute, Melbourne, VIC, Australia. (elle.martin@mcri.edu.au)

Elly Lynch. Victorian Clinical Genetics Services, Melbourne, VIC, Australia. (ella.lynch@vcgs.org.au)

Emma Krzesinski. Monash Health, Melbourne, VIC, Australia. (elizabeth.palmer@unsw.edu.au)

Elizabeth E. Palmer. Sydney Children's Hospital Network, Sydney, NSW, Australia. (elizabeth.palmer@unsw.edu.au)

Esther Pierini. Murdoch Children's Research Institute, Melbourne, VIC, Australia. (esther.pierini@mcri.edu.au)

Evanthia O. Madelli. Murdoch Children's Research Institute, Melbourne, VIC, Australia. (vana.madelli@mcri.edu.au)

Francisco Santos Gonzalez. The University of Melbourne, Melbourne, VIC, Australia. (fsantosgonza@student.unimelb.edu.au)

Gareth Baynam. Undiagnosed Diseases Program, Genetic Health WA and Rare Care Centre, Perth Children's Hospital. (gareth.baynam@health.wa.gov.au)

Gunjan Garg. Genetics of Learning Disability Service, Newcastle, NSW, Australia. (gunjan.garg@health.nsw.gov.au)

Hamish Scott. SA Pathology, Adelaide, SA, Australia. (hamish.scott@sa.gov.au)

Hannah Thomson. Genetics of Learning Disability Service, Newcastle, NSW, Australia. (hannah.thomson1@health.nsw.gov.au)

Himanshu Goel. Genetics of Learning Disability Service, Newcastle, NSW, Australia. (himanshu.goel@health.nsw.gov.au)

Ilias Goranitis. Murdoch Children's Research Institute, Melbourne, VIC, Australia. (ilias.goranitis@mcri.edu.au)

Ira W. Deveson. The Garvan Institute of Medical Research, Sydney, NSW, Australia. (i.deveson@garvan.org.au)

Isabella Pfundt. Monash Health, Melbourne, VIC, Australia. (Isabella.Pfundt@monashhealth.org)

Jacqui Russell. Sydney Children's Hospital Network, Sydney, NSW, Australia. (jacqui.russell@health.nsw.gov.au)

Janine Smith. Sydney Children's Hospital Network, Sydney, NSW, Australia. (janine.smith@health.nsw.gov.au)

Jason Pinner. Sydney Children's Hospital Network, Sydney, NSW, Australia. (jason.pinner@health.nsw.gov.au)

John Christodoulou. Murdoch Children's Research Institute, Melbourne, VIC, Australia. (john.christodoulou@mcri.edu.au)

Julia R. Broadbent. Murdoch Children's Research Institute, Melbourne, VIC, Australia. (broadbentj@student.unimelb.edu.au)

Julie McGaughran. Genetic Health Queensland, Royal Brisbane & Women's Hospital, Brisbane, QLD, Australia. (julie.mcgaughran@health.qld.gov.au)

Karin Kassahn. SA Pathology, Adelaide, SA, Australia. (karin.kassahn@sa.gov.au)

Katherine Lewis. Sydney Children's Hospital Network, Sydney, NSW, Australia. (katherine.lewis@health.nsw.gov.au)

Katrina Bell. Murdoch Children's Research Institute, Melbourne, VIC, Australia. (katrina.bell@mcri.edu.au)

Kaustuv Bhattacharya. Sydney Children's Hospital Network, Sydney, NSW, Australia. (kaustuv.bhattacharya@health.nsw.gov.au)

Kirsten Boggs. Sydney Children's Hospital Network, Sydney, NSW, Australia. (kirsten.boggs@health.nsw.gov.au)

Kirsty West. Genomic Medicine, Royal Melbourne Hospital, Melbourne, VIC, Australia. (Kirsty.West@mh.org.au)

Kristi Jones. Sydney Children's Hospital Network, Sydney, NSW, Australia. (kristi.jones@health.nsw.gov.au)

Laura Wedd. Centre for Population Genomics, Garvan Institute of Medical Research and UNSW Sydney, Sydney, NSW, Australia. (l.wedd@garvan.org.au)

Lauren Dreyer. Undiagnosed Diseases Program, Genetic Health WA and Rare Care Centre, Perth Children's Hospital. (lauren.dreyer@health.wa.gov.au)

Leah Frajman. Murdoch Children's Research Institute, Melbourne, VIC, Australia. (leah.frajman@mcri.edu.au)

Leanne Baxter. Pathology Queensland, Royal Brisbane and Women's Hospital, Herston, QLD 4029, Australia. (leanne.baxter2@health.qld.gov.au)

Lilian Downie. Victorian Clinical Genetics Services, Melbourne, VIC, Australia. (lilian.downie@vcgs.org.au)

Lily Loughman. Undiagnosed Diseases Program, Genetic Health WA and Rare Care Centre, Perth Children's Hospital. (Lily.Loughman@health.wa.gov.au)

Lisa Bristowe. Sydney Children's Hospital Network, Sydney, NSW, Australia. (lisa.bristowe@health.nsw.gov.au)

Lisa Ewans. Sydney Children's Hospital Network, Sydney, NSW, Australia. (lisa.ewans@health.nsw.gov.au)

Louise Cilento. Women's and Children's Hospital, Adelaide, SA, Australia. (louise.cilento@sa.gov.au)

Lucy Kevin. Sydney Children's Hospital Network, Sydney, NSW, Australia. (lucy.kevin@health.nsw.gov.au)

Lyndal Douglas. Sydney Children's Hospital Network, Sydney, NSW, Australia. (lyndal.douglas@health.nsw.gov.au)

Madeleine Harris. Murdoch Children's Research Institute, Melbourne, VIC, Australia. (madeleine.harris@mcri.edu.au)

Maie Walsh. Genomic Medicine, Royal Melbourne Hospital, Melbourne, VIC, Australia. (Maie.Walsh@mh.org.au)

Manisha Chauhan. Sydney Children's Hospital Network, Sydney, NSW, Australia. (Manisha.chauhan1@health.nsw.gov.au)

Margit Shah. Sydney Children's Hospital Network, Sydney, NSW, Australia. (margit.shah@health.nsw.gov.au)

Martin Delatycki. Victorian Clinical Genetics Services, Melbourne, VIC, Australia. (martin.delatycki@vcgs.org.au)

Mathew Wallis. Tasmanian Clinical Genetics Service, Hobart, TAS, Australia. (mathew.wallis@ths.tas.gov.au)

Matthew Hunter. Monash Health, Melbourne, VIC, Australia. (matthew.hunter@monashhealth.org)

Megan Ball. Victorian Clinical Genetics Services, Melbourne, VIC, Australia. (megan.ball@vcgs.org.au)

Megan Higgins. Genetic Health Queensland, Royal Brisbane & Women's Hospital, Brisbane, QLD, Australia. (megan.higgins@health.qld.gov.au)

Meutia Kumari. The Garvan Institute of Medical Research, Sydney, NSW, Australia. (michael.fahey@monash.edu)

Michael Fahey. Monash Health, Melbourne, VIC, Australia. (michael.fahey@monash.edu)

Michael Field. Genetics of Learning Disability Service, Newcastle, NSW, Australia. (Mike.Field@health.nsw.gov.au)

Mohammadreza Hajjari. Centre for Population Genomics, Garvan Institute of Medical Research and UNSW Sydney, Sydney, NSW, Australia. (mohammadreza.hajjari@populationgenomics.org.au)

Molly Carr. Sydney Children's Hospital Network, Sydney, NSW, Australia. (molly.carr@health.nsw.gov.au)

Monique Dunstan. Murdoch Children's Research Institute, Melbourne, VIC, Australia. (monique.dunstan@mcri.edu.au)

Natalie Stewart. Victorian Clinical Genetics Services, Melbourne, VIC, Australia. (natalie.stewart@vcgs.org.au)

Natalie Tan. Victorian Clinical Genetics Services, Melbourne, VIC, Australia. (natalie.tan@vcgs.org.au)

Natasha Brown. Victorian Clinical Genetics Services, Melbourne, VIC, Australia. (natasha.brown@vcgs.org.au)

Nicole Van Bergen. Murdoch Children's Research Institute, Melbourne, VIC, Australia. (nicole.vanbergen@mcri.edu.au)

Noelia Nunez-Martinez. Sydney Children's Hospital Network, Sydney, NSW, Australia. (noelia.nunezmartinez@health.nsw.gov.au)

Oliver Heath. Victorian Clinical Genetics Services, Melbourne, VIC, Australia. (oliver.heath@vcgs.org.au)

Rachel Austin. Genetic Health Queensland, Royal Brisbane & Women's Hospital, Brisbane, QLD, Australia. (rachel.austin@health.qld.gov.au)

Rani Sachdev. Sydney Children's Hospital Network, Sydney, NSW, Australia. (rani.sachdev@health.nsw.gov.au)

Rebecca Macintosh. Sydney Children's Hospital Network, Sydney, NSW, Australia. (rebecca.macintosh@health.nsw.gov.au)

Rebecca Vink. Sydney Children's Hospital Network, Sydney, NSW, Australia. (Rebecca.Vink@health.nsw.gov.au)

Rocio Rius. Centre for Population Genomics, Garvan Institute of Medical Research and UNSW Sydney, Sydney, NSW, Australia. (rocio.rius@populationgenomics.org.au)

Ruvi Samarasekera. Sydney Children's Hospital Network, Sydney, NSW, Australia. (Ruvishani.Samarasekera@health.nsw.gov.au)

Ryan Pysar. Genetics of Learning Disability Service, Newcastle, NSW, Australia. (ryan.pysar@health.nsw.gov.au)

Sandra Cooper. Sydney Children's Hospital Network, Sydney, NSW, Australia. (sandra.cooper@sydney.edu.au)

Sarah Casauria. Murdoch Children's Research Institute, Melbourne, VIC, Australia. (sarah.casauria@mcri.edu.au)

Sarah Collinson. Victorian Clinical Genetics Services, Melbourne, VIC, Australia. (sarah.collinson@vcgs.org.au)

Sarah Jelenich. Murdoch Children's Research Institute, Melbourne, VIC, Australia. (sarah.jelenich@mcri.edu.au)

Sarah Josephi-Taylor. Sydney Children's Hospital Network, Sydney, NSW, Australia. (sarah.josephitaylor@health.nsw.gov.au)

Sarah Sandaradura. Sydney Children's Hospital Network, Sydney, NSW, Australia. (sarah.sandaradura@health.nsw.gov.au)

Sean Massey. Murdoch Children's Research Institute, Melbourne, VIC, Australia. (sean.massey@mcri.edu.au)

Shannon LeBlanc. Women's and Children's Hospital, Adelaide, SA, Australia. (shannon.leblanc@sa.gov.au)

Shuxiang Goh. Sydney Children's Hospital Network, Sydney, NSW, Australia. (Shuxiang.Goh@health.nsw.gov.au)

Simon Bodek. Austin Hospital, Melbourne, VIC, Australia. (Simon.BODEK@austin.org.au)

Simon Sadedin. Murdoch Children's Research Institute, Melbourne, VIC, Australia. (simon.sadedin@mcri.edu.au)

Simran Kaur. Murdoch Children's Research Institute, Melbourne, VIC, Australia. (simran.kaur@mcri.edu.au)

Smitha Kumble. Victorian Clinical Genetics Services, Melbourne, VIC, Australia. (smitha.kumble@vcgs.org.au)

Susan M. White. Victorian Clinical Genetics Services, Melbourne, VIC, Australia. (sue.white@vcgs.org.au)

Suzanne Sallevelt. Women's and Children's Hospital, Adelaide, SA, Australia. (suzanne.sallevelt@sa.gov.au)

Tegan Stait. Murdoch Children's Research Institute, Melbourne, VIC, Australia. (tegan.stait@mcri.edu.au)

Tian Zhao. Murdoch Children's Research Institute, Melbourne, VIC, Australia. (tian.zhao@vcgs.org.au)

Tiffany Boughtwood. Murdoch Children's Research Institute, Melbourne, VIC, Australia. (tiffany.boughtwood@health.gov.au)

Tim Sikora. Murdoch Children's Research Institute, Melbourne, VIC, Australia. (tim.sikora@mcri.edu.au)

Timo Lassmann. The Kid's Research Institute, Perth, WA, Australia. (Timo.Lassmann@thekids.org.au)

Tiong Yang Tan. Murdoch Children's Research Institute, Melbourne, VIC, Australia. (tiong.tan@vcgs.org.au)

Tracy Dudding-Byth. Genetics of Learning Disability Service, Newcastle, NSW, Australia. (tracy.dudding@health.nsw.gov.au)

Michelle de Silva. Victorian Clinical Genetics Services. (michelle.desilva@vcgs.org.au)

Simone Tregoning. Victorian Clinical Genetics Services. (simone.tregoning@vcgs.org.au)

Chirag Patel. Genetic Health QLD. (chirag.patel2@health.qld.gov.au)

Ignatius Menzies. The Garvan Institute of Medical Research, Sydney, NSW, Australia. (i.menzies@garvan.org.au)

Noha Elserafy. Sydney Children's Hospital. (noha.elserafy@health.nsw.gov.au)

Jonathan Rodgers. Genetic Health Queensland. (Jonathan.Rodgers@health.qld.gov.au)

Genevieve Say. Tasmanian Clinical Genetics Service. (genevieve.say@ths.tas.gov.au)

Swaha Bose. Genetic Health Queensland. (swaha.bose@health.qld.gov.au)
